# Bioleaching of critical trace metals by *Sphingomonas desiccabilis*: substrate-driven selectivity in Earth and space analogues

**DOI:** 10.3389/fmicb.2026.1741305

**Published:** 2026-04-10

**Authors:** Luca Tonietti, Mattia Esposito, Mirko Leggiero, Fiona Bunn, Lorna J. Eades, Angelina Cordone, Louise Horsfall, Giovanni Covone, Charles S. Cockell, Donato Giovannelli, Alessandra Rotundi, Rosa Santomartino

**Affiliations:** 1 UK Centre for Astrobiology, School of Physics and Astronomy, University of Edinburgh, Edinburgh, United Kingdom; 2 Department of Science and Technology, Universitá di Napoli Parthenope, Naples, Italy; 3 Department of Biology, Universitá degli Studi di Napoli “Federico II”, Naples, Italy; 4 INAF-OAC, Osservatorio Astronomico di Capodimonte, Naples, Italy; 5 School of Biological Sciences, University of Edinburgh, Edinburgh, United Kingdom; 6 School of Chemistry, University of Edinburgh, Edinburgh, United Kingdom; 7 National Research Council, Institute of Marine Biological Resources and Biotechnologies, CNR-IRBIM, Ancona, Italy; 8 Department of Marine and Coastal Science, Rutgers University, New Brunswick, NJ, United States; 9 Marine Chemistry and Geochemistry Department, Woods Hole Oceanographic Institution, Falmouth, MA, United States; 10 Earth-Life Science Institute, ELSI, Tokyo Institute of Technology, Tokyo, Japan; 11 Department of Biological and Environmental Engineering, Riley-Robb Hall, College of Agriculture and Life Sciences, Cornell University, Ithaca, NY, United States

**Keywords:** biomining, cerium, ISRU, rare earth elements (REE), space biology, space biotechnology, *Sphingomonas desiccabilis*, thorium

## Abstract

**Background:**

Biotechnological advances are transforming the potential for sustainable resource utilization in space exploration. Biomining, using microorganisms to extract valuable metals, has emerged as a viable strategy for in *situ* resource utilization (ISRU) in extraterrestrial environments. However, an increasing body of literature is showing that selecting the most efficient bioleaching approach highly depends on the interaction between the microbial species, the type of rock and environmental conditions. In the space biomining context, the heterotrophic bacterium *Sphingomonas desiccabilis* has demonstrated its capacity to extract valuable metals in space.

**Methods:**

This study harnessed a comparative bioleaching analysis to investigate the organism’s ability to extract industrially relevant metals from seven terrestrial and extraterrestrial rock types, including a meteorite, a basaltic rock, and samples from the Sudbury Basin, an impact structure formed ~1.85 Ga ago.

**Results:**

We demonstrated that, depending on the substrate mineralogy, *S. desicca bilis* selectively mobilized Ce (up to 10.6%), Th (up to 1.5%), and other metals such as Pd, Pt, Mo, and W, at circum-neutral pH conditions. However, the extraction was not equal across all substrates, indicating the importance of mineralogy in bioleaching.

**Discussion:**

While extraction rates were lower compared to industrial biomining standards, these results underscore the organism’s potential in low-grade rocks bioleaching, particularly relevant for sustainable terrestrial biomining and long-duration space missions. More broadly, this work demonstrates that bioleaching efficiency is shaped not only by the microbial species employed, but also by the specific characteristics of the substrate, emphasizing the need to tailor bioleaching strategies to distinct mineral contexts.

## 1 Introduction

Biomining is the application of biotechnology to the field of mining and resource extraction, utilizing microbial activities to extract metals from ores and low-grade minerals. Its wide use on terrestrial settings offers various advantages over traditional mining practices, including lower energy consumption, reduced environmental impact, and the ability to extract metals from economically unviable low-grade ore and electronic waste (Kaksonen et al., 2020). This method exploits the unique metabolic capabilities of some microorganisms, alone or in consortia, to facilitate the dissolution and recovery of metals from rocky substrates (Habibi et al., 2020). Beyond Earth, extraterrestrial *in situ* mining has emerged as a promising approach for unlocking long-term space exploration, offering potential solutions to resource limitation challenges (Linne et al., 2017; Cockell et al., 2020). *In Situ* Resource Utilization (ISRU) involves extracting and processing resources on-site, reducing the reliance of terrestrial resources transportation in the frame of long-duration space missions, thus improving their sustainability (Sanders and Larson, 2013). Biomining in particular has garnered attention as a promising technique for resource extraction from rocks in extraterrestrial environments and ISRU (Gumulya et al., 2022; Santomartino et al., 2022). Indeed, microbial systems offer advantages such as operating under extreme conditions, e.g., extremotolerants and extremophiles, enhancing metal extraction rates from low-grade ores, lowering costs and minimising environmental footprint (Haferburg et al., 2017; Merino et al., 2019; Gumulya et al., 2022; Murthy et al., 2022). Understanding the interactions between microorganisms and their mineralogical and geochemical environment under space conditions is crucial for optimizing biomining strategies in extraterrestrial settings.

Single species as well as microbial consortia play a vital role in biomining by catalyzing chemical reactions that lead to metal solubilization through different mechanisms, e.g., oxidation, reduction, and complexation of ions (Rawlings and Johnson, 2007). Iron-oxidizing chemolithotrophic microorganisms, e.g., *Acidithiobacillus ferrooxidans, Leptospirillum ferriphilum, Metallosphaera sedula*, have traditionally played a significant role in biomining processes by catalyzing the oxidation of ferrous iron and facilitating the solubilization of valuable metals from sulfidic ores (Arshadi and Yaghmaei, 2020; Farías et al., 2021; Adetunji et al., 2023; Tonietti et al., 2023, 2024), achieving metal recovery rates ≥80–90% under acidic conditions (pH < 2) from high-grade sulfide ores, through the use of consortia that sustain Fe and S redox cycling (León et al., 2025). In a space biomining context, using chemolithotrophic leaching offers the advantage of not requiring an organic nutrient source. However, its dependence on sulfur-bearing minerals renders it infeasible on planetary bodies such as the Moon, many asteroids and even some regions of Mars (Cockell and Santomartino, 2022; Santomartino et al., 2024). Beside chemolithotrophs, heterotrophic bacteria, fungi and archaea, have demonstrated their capacity to participate in diverse mineral transformation reactions and metal-leaching processes (Bindschedler et al., 2017). Some heterotrophic microorganisms can perform bioleaching by producing metabolites and organic acids that promote mineral dissolution and metal solubilization (Chaerun et al., 2017), biosorption (Tangaromsuk et al., 2002), or through enzymatic activity. For instance, oxidoreductases such as multicopper oxidases, monooxygenases and dehydrogenases can mediate redox transformations of metal-bearing minerals, enhancing metal mobilization, while metal-binding enzymes and siderophore-associated pathways may facilitate complexation and solubilization of trace elements (Shah et al., 2022; Hay Mele et al., 2023). Besides bioleaching mechanisms for single species cultures, industrial biomining frequently employs microbial consortia due to metabolic complementarity and improved elemental cycling (Rawlings and Johnson, 2007; Lane et al., 2025).

*Sphingomonas desiccabilis*, a heterotrophic mesophilic microorganism, has gained attention for its potential role in supporting space exploration (Cockell et al., 2020; Santomartino et al., 2022), particularly for its capacity to extract elements from low-grade rocks, which are relevant in space setting (Cockell et al., 2020, 2021). *S. desiccabilis* demonstrated capabilities in extracting industrially significant metals, such as rare earth elements (REEs) and V, from Icelandic basalt (a low-grade rock) onboard the International Space Station (ISS), and various other elements (K, Mn, Fe, Ni, Sr., Zr, and Mo) from a L-chondrite meteorite (Cockell et al., 2020, 2021; Santomartino et al., 2024; Santomartino et al., 2026) under terrestrial gravity conditions, making it a promising candidate for space biomining. *Sphingomonas* spp. isolated from mine tailings were able to extract Ag and Mn from electronic waste (printed circuit boards, Díaz-Martínez et al., 2019; Huerta-Rosas et al., 2020). However, *S. desiccabilis* capacity to extract Mg and Ni from ultramafic rocks was lower compared to other heterotrophic species (Plante et al., 2025). All these findings highlight that, particularly for future space applications, biomining outcomes might vary substantially depending on the microbial species, mineral substrate, and environmental conditions, underscoring the need to carefully match microbial systems to specific resource targets in space applications (Santomartino et al., 2024, 2026). In this context, *S. desiccabilis* biomining performance appears promising although strongly dependent on both substrate mineralogy and experimental conditions.

In this work, we performed a systematic comparison of *S. desiccabilis* bioleaching performance across mineralogically diverse, space-relevant terrestrial and extraterrestrial substrates, to understand its potential relevance for substrate-tailored biomining strategies, applicable to both terrestrial low-grade ores and future space-based ISRU operations.

## 2 Materials and methods

### 2.1 Microbial species and growth conditions

We used the bacterium *Sphingomonas desiccabilis* CP1D (DSMZ), a Gram negative, non-motile, non-spore-forming desiccation-resistant microorganism first isolated from the Colorado Plateau (Reddy and Garcia-Pichel, 2007). This species was selected for its demonstrated capacity to mobilize REEs, V and other metals from basaltic and meteoritic (L-chondrite) rocks under different gravity conditions onboard the ISS (Cockell et al., 2020, 2021; Santomartino et al., 2026). Beyond this, *S. desiccabilis* exhibits several physiological traits relevant to space bioleaching applications, such as desiccation tolerance, biofilm formation, extracellular polymeric substances (EPS) production, which facilitate surface attachment and microbe-mineral interface interactions (Cockell et al., 2020, 2021; Stevens et al., 2019).

The standard minimal medium M9 was selected for this experiment due to its defined mineral composition and limited nutritional content, which was hypothesized to encourage the microorganisms to use the rock substrate as a nutrient source. M9 medium composition was: 6.00 g/L of Na_2_HPO_4_, 3.00 g/L of KH_2_PO_4_, 0.50 g/L of NaCl, 1.00 g/L of NH_4_Cl, 100 μL of 1 M MgSO_4_, 100 μL 1 M CaCl_2_, and 2% m/V glucose as a carbon source. Overnight starting cultures grew in M9 medium at 25 °C under static conditions for ~48 h until reaching exponential phase (OD_600_ = ~0.2). Cultures were subsequently diluted in fresh sterile M9 medium to obtain the required starting OD_600_ for bioleaching experiments.

R2A solid medium was used for the Colony Forming Unit (CFU) assay (Section 2.5). Its composition was 0.50 g/L yeast extract, 0.50 g/L protease peptone, 0.50 g/L casein hydrolysate, 0.50 g/L glucose, 0.50 g/L soluble starch, 0.30 g/L sodium pyruvate, 0.30 g/L K_2_HPO_4_, 0.024 g/L MgSO_4_ anhydrous and, 15.0 g/L agar-agar.

### 2.2 Substrate selection and preparation

The rocks used as substrates for the bioleaching experiment (Section 2.5) are listed in Table 1.

**Table 1:** Rocks, and their origin, used as substrates in our experiments.

Rock type	Abbreviation	Origin
Basalt	BAS	Iceland (Loudon et al., 2018; Santomartino et al., 2020; Cockell et al., 2020, 2021)
Eucrite	EU	Skylab (US)
05AV08	05AV08	Levack Mine, Sudbury Basin, Canada
L11	L11	Levack Mine, Sudbury Basin, CanadaLevack Mine, Canada
05AV30	05AV30	McCreedy Mine, Sudbury Basin, Canada
CC1	CC1	Creighton Mine, Sudbury Basin, Canada
CA4	CA4	Creighton Mine, Sudbury Basin, Canada

Substrates were selected to reflect both terrestrial and space-relevant lithologies, including: (1) Five sulfide-rich mine rocks from the Sudbury Basin impact structure (~1.85 Ga) (Abramov and Kring, 2004; Riller, 2005), whose impact-derived textures and mineralogy are analogous to the shocked, glass-rich, and brecciated materials prevalent on the Moon and Mars (Stoeffler et al., 1979; Crandall et al., 2021; Harrison et al., 2025), (2) basaltic material representative of lunar and Martian crust (BAS), and (3) an extraterrestrial eucrite (EU). This diversity allowed the evaluation of substrate-driven selectivity across sulfide- and Si-dominated matrices. Samples 05AV08 and L11 originate from the Levack Mine, 05AV30 from the McCreedy Mine, and CC1 and CA4 both are from the Creighton Mine, Sudbury Basin. BAS is the same type of basaltic rock used in the BioRock experiments on the ISS (Loudon et al., 2018; Cockell et al., 2020, 2021), while EU is an achondrite stony basaltic meteorite from the crust of 4 Vesta (Shisseh et al., 2024).

To increase the surface available for microbial extraction, the rocks were crushed by hammering and subsequently sorted using a sieve with a mesh size of 1.00 mm, using fragments ≤1.00 mm as substrates for the experiments, to increase the available surface area. An amount of 0.15 g of fragments from each rock type was transferred into individual 25.00 mL glass tubes, each representing a single experimental replicate (for each rock: 3 biological replicates, 1 abiotic control). The rock-containing tubes were subsequently sterilized by dry-heating at 250 °C for 4 h in a hot air oven (Carbolite Type 301, UK). These conditions are below the thermal transformation thresholds of the dominant mineral phases present in these substrates, including silicates (plagioclase, pyroxene, olivine, 600–800 °C), oxides, and primary sulfides (chalcopyrite, pyrrhotite, pentlandite, 400–600 °C), therefore significant modification of the key geochemical properties is not expected.

### 2.3 Substrate preliminary characterization

#### 2.3.1 X-ray diffraction (XRD)

XRD analysis of the substrates was performed at the School of Geosciences, University of Edinburgh, as described in Santomartino et al. (2024). Briefly, each selected rock (*n* = 3) was carefully crushed in a mortar and pestle into a powder and weighted (~ 1 g each). The powder samples were fed into a Bruker D8-Advance X-ray Diffractometer, using a 2-theta configuration in which the X-rays were generated by a Cu-anode X-ray tube operating at 40 kV and a tube current of 40 mA. Diffracted X-rays were detected using a sodium iodide scintillation detector. The samples were scanned from 2 to 60° two theta with a scan rate of 0.02° per second. Resultant diffractograms were compared to the International Centre for Diffraction Data (ICDD) diffractogram database library (2012 issue) using the EVA analysis package. Typically, this procedure gives a detection limit for crystalline phases of approximately 1 w/t %. Mineral abundances were analysed by Rietveld analysis using the TOPAS software package.

#### 2.3.2 ICP-MS/ICP-OES geochemical analysis of the rocks

Inductively Coupled Plasma Mass Spectrometry (ICP-MS) and Inductively Coupled Plasma Optical Emission Spectroscopy (ICP-OES) were used to determine the elemental concentrations in the various rock types. Preparation procedure of the samples prior to analysis was as follows: roughly 25–50 mg of powdered and homogenized rock was added to Savillex Teflon vessels [*n* = 3, except for BAS *n* = 2; for BAS, further rock characterization can be found in Loudon et al. (2018)]. Rock standards (Georem standards BCR2, BHVO1 and B-EN) were prepared in the same way. For the total digestion of these samples, 3 mL of double distilled HNO_3_, 2 mL HCl and 0.8 mL HF was added to each of the vessels. HF was added after the other acids to prevent disassociation, formation and precipitation of aluminum fluorides. The HF addition is a necessary step in this protocol, however it compromises the detection of silicon from the rocks, due to its volatilization. Samples were placed on a hot plate for digestion overnight at 120 °C and checked for complete digestion. Following this, they were then evaporated on the hot plate. Five milliliter of 1 M HNO_3_ was added to each vessel which were then closed and returned to the hot plate for a second digestion step. Samples were further diluted with 2–5% (v/v) HNO_3_ for ICP-OES and ICP-MS analyses. An agilent Vista Pro ICP-OES was used to measure major and minor elements in the samples. The ICP-OES results are reported in mg per gram of chondrite. For the trace elements, the analysis was carried out on a high resolution, sector field, ICP-MS (Nu AttoM). The ICP-MS measurements were performed in low resolution (300), in Deflector jump mode with a dwell time of 1 ms and 3 cycles of 500 sweeps. Data were reported in micrograms of elements per gram of chondrite.

### 2.4 Bioleaching experiment

For each replicate, 0.15 g of each substrate was placed in a 25.0 mL glass tube (as detailed in 2.2). Fifteen mL of M9 sterile medium were added to each tube, reaching a final pulp density of 1% (w/v). An appropriate volume V_i_ of a *S. desiccabilis* overnight culture (OD_600_ ~ 0.2) to achieve a homogeneous starting OD_600_ of 0.039 ± 0.006 across samples was inoculated into each rock-containing tube (*n* = 3 per rock), except for abiotic controls (rock + medium without microbial inoculation; *n* = 1 per rock). The inoculum volume V_i_ was calculated using the classic formula V_i_ = (*C_f_* × V_f_)/C_i_, where C_i_ corresponds to the OD_600_ of the starting overnight culture, *C_f_* is OD_600_ = 0.039, and V_f_ is the final culture volume of 15.0 mL. Fifteen milliliter of a *S. desiccabilis* culture without substrate (*n* = 7) was also prepared as a positive control (i.e., microbial growth in the absence of rock). Samples were incubated for 30 days statically without shaking at 25 °C. Static incubation was intentionally selected to: (i) favor biofilm formation at the microbe-mineral interface; and (ii) simulate low-energy conditions relevant to potential ISRU applications, where continuous agitation may not be feasible. The pH of the liquid cultures was measured before and after the incubation (day 0 and day 30).

At each time point (day 0 and 30), microbial growth was assessed by 2 complementary methods. Before both analysis, cultures were gently mixed by gentle tube shaking and culture pipetting to resuspend planktonic cells and loosely attached biomass without mechanically disrupting rock-associated biofilms, improving sample homogeneity. To assess microbial concentration, 100 μL aliquot of the cultures was collected to measure optical density at a wavelength of 600 nm (OD_600_), using fresh M9 medium as a blank. The colony forming unit (CFU) assay was performed to measure microbial cell viability in the presence of different substrates. An aliquot of each culture was collected and serially diluted in a 1:10 series to obtain eight total dilutions. The final four dilutions (10^−5^, 10^−6^, 10^−7^, 10^−8^) were plated on R2A solid medium for CFU enumeration. Plates were sealed with parafilm and placed at 20 °C until colonies were visible (~ 3 days). Single colonies were counted and the corresponding dilution factor was recorded. The CFU/mL was calculated using the formula CFU/mL = (Total colonies) × (dilution factor)/volume, where volume corresponded to the volume of culture used for that specific dilution (50 μL).

### 2.5 Preparation of biological samples for scanning electron microscopy (SEM)

Samples were fixed in 3% (v/v) glutaraldehyde in 10 mM HEPES buffer (pH 7.0) at 4 °C for 5 days to preserve cell morphology. Following fixation, samples were dehydrated in ethanol solution at increasing percentages [10–100% (v/v), 10 min per step] at room temperature. Dehydrated samples were stored at 4 °C prior to drying using liquid CO₂ in a Polaron E3100 critical point dryer. Samples were mounted on aluminum stubs (Agar Scientific) using carbon adhesive tabs (Agar Scientific), sputter-coated with a 0.2 nm gold layer (Denton Vacuum), and examined using a Zeiss SIGMA HD VP field emission SEM at 15 kV under various magnifications.

### 2.6 Fluorescence microscopy

At the end of the experiment, a portion of the rock sediment was treated with 1% (v/v) formaldehyde solution and stored at 4 °C until microscopy analysis, to preserve the biofilm. Prior to staining, the formaldehyde solution was carefully removed by gentle pipetting, and the sediment was gently washed twice with sterile phosphate-buffered saline (PBS) to remove residual fixative. The samples were then treated with 1 mL of Sybr Gold (Thermo Fisher) diluted 1:10,000. Samples were shielded from light by covering them with aluminum foil, and were incubated for 30 min at room temperature (~ 20 °C). The staining solution was then carefully removed, and excess was left to dry for 5–10 min at room temperature. The sediment was then cautiously collected and transferred onto microscope slides for biofilm visualization using a Leica DM4000 B fluorescent microscope, with a 40x magnification objective (Leica) and blue light (I3 prism).

### 2.7 Biofilm quantification with crystal violet

In addition to the microscopic techniques (2.6, 2.7), biofilm was quantified using a crystal violet (CV) assay. For each culture from the bioleaching experiment, a portion of the rock powder was placed in 1.5 mL tubes. The sediment was subjected to three series of delicate washing steps using 1 mL of phosphate-buffered saline (PBS). Care was taken during pipetting to ensure minimal disturbance to the biofilm structure. Subsequently, the biofilms were stained with a 1 mL solution of CV 0.4% w/v for 30 min at room temperature. Following the staining period, the CV solution was carefully removed and the rock powder with biofilm was washed again 3 times with 1 mL of PBS. The final volume of PBS was removed, and 1 mL of 33% v/v acetic acid (destain solution) was added to remove the residual CV attached to the biofilm. For each sample, a volume of 100 μL of the destain solution was transferred to an optical 96-well plate with a flat bottom (Corning). Optical density readings were obtained at a wavelength of 570 nm (OD_570_), using 33% (v/v) acetic acid solution as a blank. For the positive controls (bacterium without the rock), biofilm quantification reflects the typical biofilm and EPS produced by *S. desiccabilis* even in absence of a substrate (Reddy and Garcia-Pichel, 2007), rather than surface-attached mineral biofilm. For these samples, 100 μL of the culture were centrifuged for 1 min at 4000 RPM to collect suspended and loosely aggregated cells. The supernatant was removed, and the resulting biomass pellet was processed as described above for the rock sediments.

### 2.8 ICP-MS and ICP-OES of the liquid fraction of the bioleaching cultures

At the end of the bioleaching experiment (day 30), 1 mL of the liquid fraction of each culture was collected and centrifuged at 5,000 rpm for 5 min, to separate the supernatant from cellular material and rock debris that could otherwise influence the leaching results. The resulting pellet was not analysed further, as biomass could not be reliably separated from rock particles. Previous studies have shown that *S. desiccabilis* biomass accounts for <5% of the total extracted metals (Cockell et al., 2020, 2021). The supernatant fraction was treated with HNO_3_ 4% (v/v) and analyzed through ICP-MS and ICP-OES to determine the total concentration of the elements from the rocks. The samples were analyzed for different elements using an Agilent 8,900 ICP-MS. Elements that were very high in concentration were analysed using a Perkin Elemer 8,300 DV ICP-OES spectrometer. Raw ICP-MS data (determined in μg/L) was converted to obtain absolute quantity of a given element in the culture chamber, taking into account dilution factors applied during ICP-MS analysis.

### 2.9 Calculation of bioleaching rates

To assess bioleaching rates, two calculations were performed: (1) Elemental extraction rate, and (2) calculation of the biotic/abiotic extraction rate.

The elemental extraction rate (1) is a measure of the % bioextraction rate of each element from each rock. It was obtained by calculating the ratio between the ICP-MS/OES of the bacterium-containing liquid fraction (Section 2.9) and the ICP-MS/OES data of each rock (Section 2.4), for each element. These values were expressed in percentage (%) by multiplying the ratios x 100.

The biotic/abiotic extraction rate (2) is a measurement of the effect that *S. desiccabil* had on the leaching, for each element and from each rock. For this measurement, the ratio between the bacterium-containing cultures and the abiotic cultures ICP-MS/OES values (Section 2.9) was calculated. For each element, an arbitrary threshold of ratio ≥2 was applied to distinguish consistent biological effect from background noise (abiotic leaching). This cutoff was chosen as a conservative criterion, ensuring that any detected effect exceeded typical analytical variability and minor experimental fluctuations (i.e., biological leaching values were at least twice those of abiotic controls).

### 2.10 Statistical analysis

All analyses were performed using open-source software in accordance with open science principles. The statistical analyses were conducted in RStudio 2023.03.0 Build 386 (R version 4.3.2) with the packages ggplot2, tidyverse, dplyr, readr, viridis, and gridExtra. Figures were prepared using RStudio and Inkscape 1.1. Data organization was managed in Google Sheets.

## 3 Results

### 3.1 Rock substrate characterization

Seven different rock substrates were used in the experiment (Table 1), including 5 from massive sulfide deposits and metalliferous mines in Canada formed within a giant impact event ~1.85 Ga ago (05AV08, 05AV30, C11, CA4, CC1), 1 basaltic rock coming from Iceland (BAS) and 1 extraterrestrial eucrite from 4 Vesta (EU). To determine the minerals and elements available for bioleaching, the rock substrates were analyzed by XRD, ICP-MS and ICP-OES.

**Figure 1:**
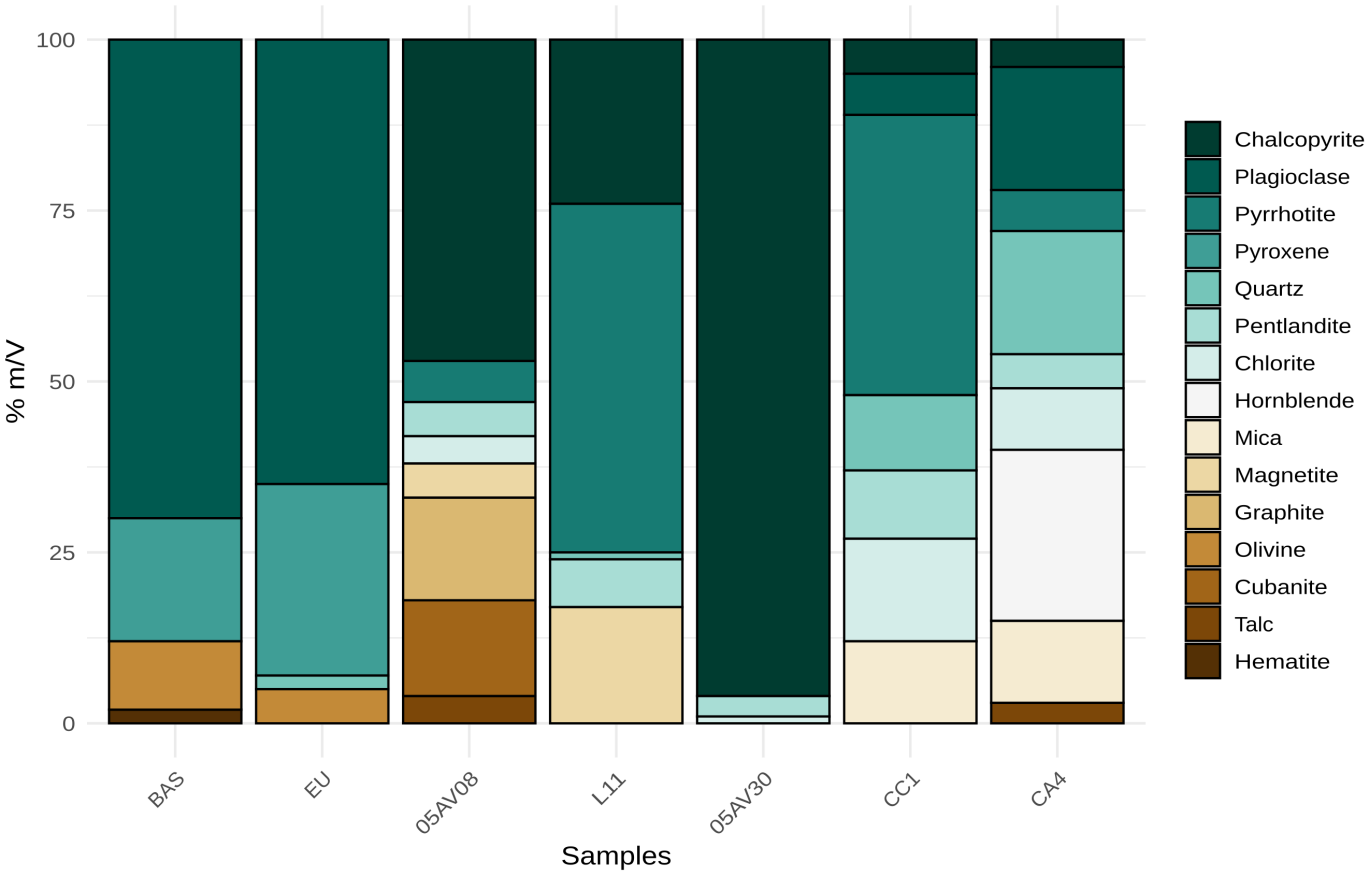
Mineralogical composition of the substrates used in this work, obtained by X-ray diffraction (XRD) analysis. Each column represents one of the seven rock samples (BAS, EU, 05AV08, L11, 05AV30, CC1, CA4). The relative abundance of minerals (% m/V) is shown as stacked bars. Colors indicate the different mineral phases identified in the samples, including major silicates (Plagioclase, Quartz, Mica, Hornblende, Pyroxene, Olivine), sulfides (Pentlandite, Pyrrhotite, Chalcopyrite, Cubanite), oxides (Magnetite, Hematite), carbon phases (Graphite), and phyllosilicates (Chlorite, Talc). Values are represented as percentages normalized to 100% within each sample.

The XRD analysis revealed significant differences in the mineralogical composition of the substrates moving from the mine samples rich in sulfides minerals to the basaltic samples rich in plagioclase [(Na, Ca)(Si, Al)_4_O_8_] and silicates (Figure 1, Table 2). Specifically, 05AV08 is the only sample that contains in percent (m/V) pure graphite 15%, chalcopyrite (CuFeS_2_) 47% and cubanite (CuFe_2_S_3_) 14% with other accessory minerals such as pyrrhotite (Fe_1-x_S, *x* = 0–0.17) 6% and pentlandite [(Ni, Fe)_9_S_8_] 5%. L11, coming from the same mine is slightly different with less mineralogical composition and an amount in % m/V of pyrrhotite 51%, chalcopyrite 24% and magnetite (Fe^2+^Fe_2_^3+^O_4_) 17% plus pentlandite 7% and quartz (SiO_2_) 1%. Both mines can be considered massive sulfide deposits due to the presence of high concentration of Fe and Cu sulfides. 05AV30 is mainly a Cu sulfide sample made by the 96% of chalcopyrite with a small amount of Ni sulfides, such as pentlandite 3% and the 1% of chlorite [(MgFeAl)_8_(SiAl)_8_O_20_(OH)_16_]. CC1 and CA4 samples, both from Creighton Mine, are richer in mineral diversity. CC1 is mainly composed of pyrrhotite 41%, with a similar amount of pentlandite, quartz, chlorite and mica [XY_2-3_Z_4_O_10_(OH)_2_, where *X* = K, Na, Ca; *Y* = Al, Mg, Fe, and *Z* = Si, Al] respectively with the 10, 11, 15, and 12% m/V. Plagioclase 6% and chalcopyrite 5% are also present. CA4 is composed by hornblende [Ca_2_(Mg, Fe, Al)_5_(Al, Si)_8_O_22_(OH)_2_] 25%, plagioclase 18%, quartz 18%, mica 12% and other accessory minerals such as chlorite 9%, pyrrhotite 6% and talc [Mg_3_Si_4_O_10_(OH)_2_] 3%. CC1 can also be considered a massive sulfide deposit due to its high presence of pyrrhotite, while CA4 is richer in hydroxides and silica minerals. BAS and EU, both basaltic-like rocks, differ in mineralogical composition compared to the other mine samples. BAS is mainly composed of plagioclase 70%, pyroxene [XY(Si, Al)_2_O_6_, where *X* = Ca, Na, Fe, Mg and *Y* = Cr, Al, Mg, Co, Mn, Sc, Ti, V) 18%, olivine [(Mg, Fe)_2_SiO_4_] 10% and a small amount of hematite (Fe_2_O_3_) 2%. EU is composed of plagioclase 65%, pyroxene 28%, olivine 5% and quartz 2%. The high abundance of olivine and pyroxene in both samples confirms their igneous origin, with little to no evidence of metamorphism or the formation of secondary minerals. The lower proportion of olivine in the EU sample may be attributed to the thinner mantle of 4 Vesta and its deep Mohorovičić discontinuity (MOHO) boundary, which influences the composition of basaltic crust material. This mineralogical profile distinguishes EU from traditional chondritic models, as noted by Clenet et al. (2014).

**Table 2:** Percentage composition (% m/V) of the main minerals found in the different rock substrates.

Mineral	BAS	EU	05AV08	L11	05AV30	CC1	CA4
Pentlandite	NA	NA	5	7	3	10	5
Pyrrhotite	NA	NA	6	51	NA	41	6
Chalcopyrite	NA	NA	47	24	96	5	4
Chlorite	NA	NA	4	NA	1	15	9
Talc	NA	NA	4	NA	NA	NA	3
Magnetite	NA	NA	5	17	NA	NA	NA
Cubanite	NA	NA	14	NA	NA	NA	NA
Graphite	NA	NA	15	NA	NA	NA	NA
Quartz	NA	2	NA	1	NA	11	18
Plagioclase	70	65	NA	NA	NA	6	18
Mica	NA	NA	NA	NA	NA	12	12
Hornblende	NA	NA	NA	NA	NA	NA	25
Hematite	2	NA	NA	NA	NA	NA	NA
Pyroxene	18	28	NA	NA	NA	NA	NA
Olivine	10	5	NA	NA	NA	NA	NA

NA indicates absence of mineral in that substrate.

The geochemical analysis of the samples, performed using ICP-MS and ICP-OES (Supplementary Table S1, Supplementary Data), revealed significant variability in elemental compositions, reflecting their diverse geological origins and mineralogical properties. Samples from the Canadian massive sulfide deposits (05AV08, L11, 05AV30, CC1, CA4) exhibited high concentrations of transition metals, consistent with their metalliferous nature. This analysis confirmed the similar composition of BAS EU, which was expected since they are both basaltic rocks. Fe, Al, Mn, Ti, and Cr dominated their composition. BAS was dominated by silicate minerals. The EU exhibits some unique geochemical profile distinct from all the terrestrial samples, such as the highest Cr, and the lowest Cu, Mo, Ni, Ru, and Zn concentration. The Levack Mine samples (AV08 and L11) exhibited high concentrations of metals such as Fe, Cu, Pz, Zn and Ni. 05AV30 from the McCreedy Mine showed the highest Cu concentration among all samples, consistent with its chalcopyrite-rich composition. Fe concentrations were slightly lower compared to the Levack samples Samples from the Creighton Mine (CA4 and CC1), exhibited geochemical characteristics more similar to the basaltic rocks BAS and EU. The most abundant rare earth element is Ce for BAS and EU, reflecting their basaltic crust origin (with slightly lower concentration in EU, highlighting a distinct geochemical signature of the asteroid-derived material), and for Creighton Mine rocks (CC1 and CA4). Tb and La were similarly abundant in CA4, which generally had a higher REEs concentration by roughly an order of magnitude compared to CC1. In AV08, L11 and AV30, REEs concentration was generally lower compared to the other rocks, although Ce was still the most abundant. Platinum group elements (PGEs) Pt, Pd and Rh were relatively abundant in sulfide-rich samples, particularly in 05AV30 and 05AV08, compared to the other rocks.

### 3.2 Microbial growth in the presence of rock substrates

Microbial growth and viability at the end of the experiment (day 30) was measured using two methods, respectively: optical density at *λ* = 600 nm (OD_600_) and colony-forming unit (CFU) assay. Statistical *t*-test allowed to compare the effect of each rock on microbial growth to the absence of substrate.

**Figure 2:**
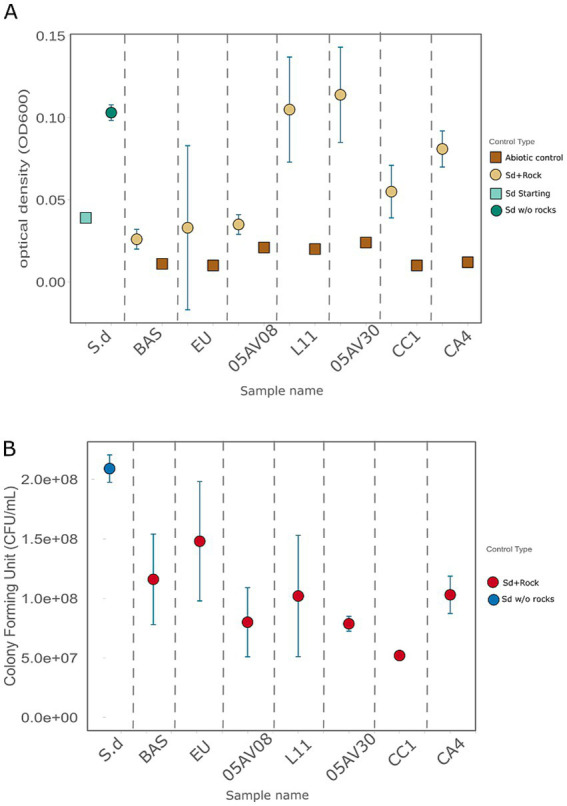
Microbial growth in the presence of the rock substrates. Samples are separated into columns representing the seven different rock substrates or the absence thereof (S.d, first column on the left). **(A)** OD_600_ of the cultures measured at the end of the experiment (day 30). Circles represent mean OD_600_ values measured for samples containing *S. desiccabilis*, either with (yellow circles) or without (dark aquamarine circle) a rock substrate; light aquamarine square represent the starting OD_600_ for all *S. desiccabilis* cultures. Brown squares represent mean OD_600_ value of abiotic samples. Error bars indicate standard errors. **(B)** Microbial viability measured as CFU/mL concentration, at the end of the experiment (day 30). Blue circle represents mean value for *S. desiccabilis* cultures in absence of rock; red circles represent mean values for cultures in the presence of a rock substrate. Error bars represent standard errors.

Optical density measurements (OD_600_) were performed to evaluate microbial growth in the presence of the 7 rock substrates or the absence thereof (Figure 2A, Supplementary Table S2). In the absence of rock, the average OD_600_ was 0.150 ± 0.015. Similar values were reached in the presence of L11 (0.105 ± 0.032, *t*-test *p*-value = 0.38) and 05AV30 (0.114 ± 0.029, *t*-test p-value = 0.43). All the other rocks reported significantly lower values (*t*-test *p*-values < 0.05) than the culture without rock. For the BAS sample, OD_600_ was 0.026 ± 0.006 (*t*-test *p*-value = 0.0002). The EU sample displayed a mean OD_600_ of 0.033 ± 0.005 (*p*-value = 0.0002), and samples containing 05AV08 had a mean OD_600_ of 0.035 ± 0.006 (*p*-value = 0.0003). CC1 showed a mean OD_600_ of 0.055 ± 0.016 (*p*-value = 0.013), and CA4 showed a mean OD_600_ of 0.081 ± 0.011 (*p*-value = 0.015). Abiotic controls (only rock) consistently showed OD_600_ values < 0.02 (Supplementary Table S2) confirming the rock did not influence the spectrophotometric readings.

We also verified the effect of the rock substrates on microbial viability, through a colony-forming unit (CFU) assay (Figure 2B, Supplementary Table S3). *S. desiccabilis* grown in the absence of rock substrate reached the highest viability, with a value of (2.09 ± 0.12) x 10^8^ CFU/mL, suggesting the addition of any rock substrate had at least a small detrimental effect on microbial survival. For BAS [(1.16 ± 0.38) x 10^8^ CFU/mL], EU [(1.48 ± 0.50) x 10^8^ CFU/mL], 05AV08 [(8.00 ± 2.97) x 10^7^ CFU/mL], and L11 [(1.02 ± 0.51) x 10^8^ CFU/mL], there was no statistically significant reduction in viability (*t*-test *p*-values > 0.05), although this could also be a consequence of the higher variability. Viability decreased in the presence of 05AV30 (*p*-value = 0.00002), CC1 (*p*-value = 0.00001) and CA4 (*p*-value = 0.11), compared to the absence of rock, with values of [(7.87 ± 0.63) x 10^7^ CFU/mL], [(5.20 ± 0.34) x 10^7^ CFU/mL] and [(1.03 ± 0.16) x 10^8^ CFU/mL] respectively.

### 3.3 Microbial interaction with the rock surface and biofilm formation

**Figure 3:**
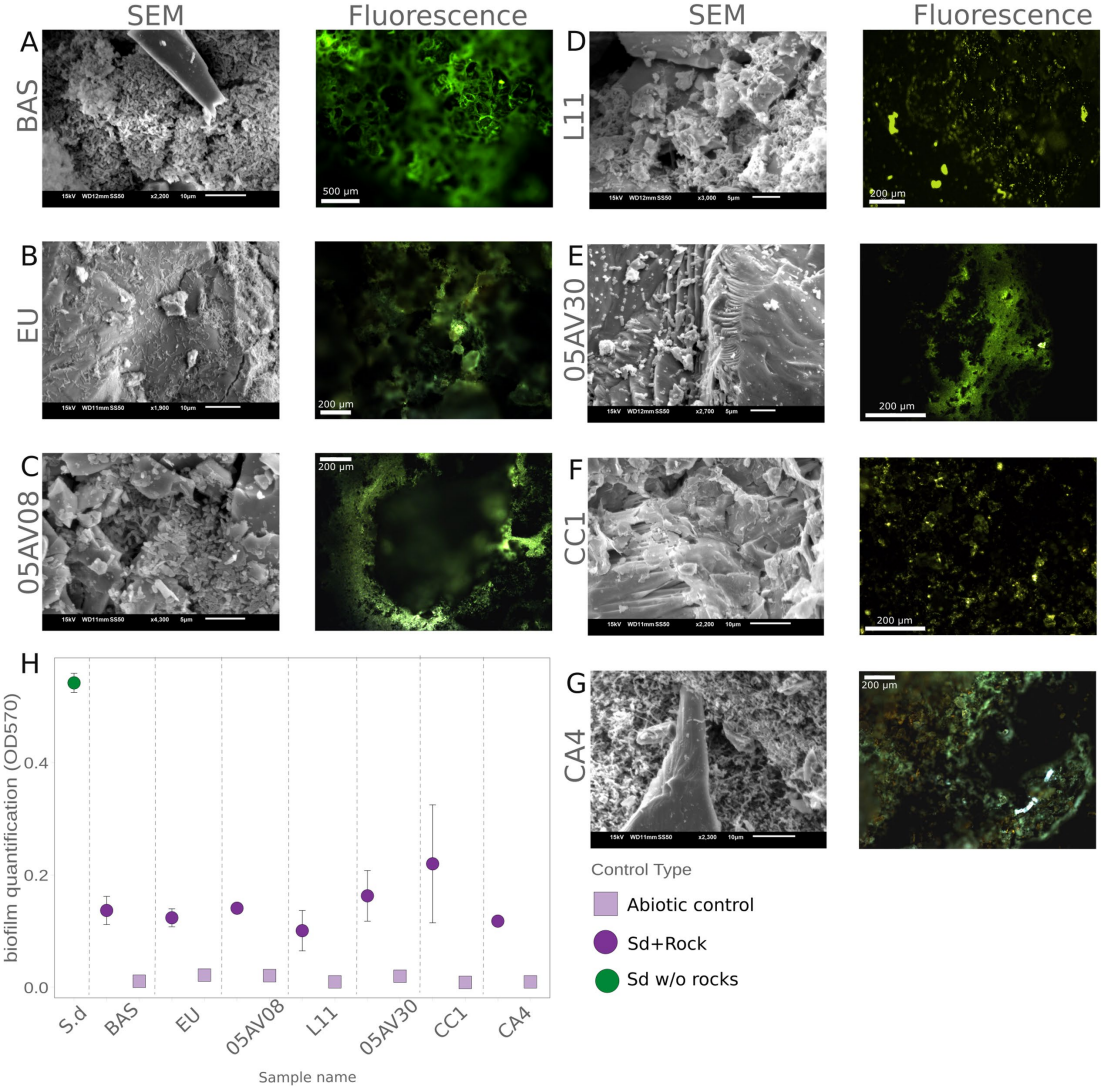
Visualization and quantification of *S. desiccabilis* biofilm formation on the rock substrates. **(A–G)** Images obtained with scanning electron microscopy (SEM; left panels) and fluorescence microscopy (right panels) are shown for each rock. For fluorescence microscopy images, biofilms were stained with Sybr-gold and are visible as green pixels. **(H)** Biofilm quantification based on crystal violet staining (OD_570_, i.e., absorbance at *λ* = 570 nm). Green circle (*S. d*) indicates mean value for *S. desiccabilis* culture without rock substrate. For each rock substrate, purple circles indicate mean values for *S. desiccabilis* cultures, and lilac squares represent abiotic samples. Error bars indicate standard errors.

To verify microbial interaction with the rock substrates, and to reveal the presence, distribution and morphology of *S. desiccabilis* biofilm, portions of the rock substrates (or an aliquot of the liquid culture for the no-rock culture control, see Section 2.8) were observed at the end of the experiment (day 30) through fluorescence microscopy (biofilm visualized through Sybr Gold staining) and scanning electron microscopy (SEM, Figures 3A–G).

In general, the presence of biofilm was evident in all analyzed samples, indicating interaction between *S. desiccabilis* and all the rock surface, although biofilm pattern and distribution varied (Figures 3A–G). The biofilm on BAS exhibited a dense network, with fluorescence intensity evenly distributed but most prominent around pores. This suggested that pores may act as aggregation sites, encouraging biofilm development (Figure 3A). A similar pattern was present for 05AV08, where moderate and even fluorescence was present around pores, although SEM showed a less dense distribution (Figure 3C). The EU sample revealed sparsely distributed microbial cells attached along the rock. The biofilm coverage was less dense compared to BAS (Figure 3B). On the L11 (Figure 3D) and CC1 (Figure 3F) substrates, fluorescence was sparse compared to other samples, with only a few small bright visible clusters. Both substrates revealed a rugged surface with scattered microbial cells. The biofilm appeared discontinuous, with clusters of cells forming along cracks in the mineral structure. In contrast, 05AV30 showed a strong fluorescence signal with a clear three-dimensional biofilm (Figure 3E). The CA4 substrate presented irregular fluorescence patterns, with areas of high intensity interspersed with dim regions, probably due to substrate heterogeneity or localized nutrient gradients (Figure 3G).

To semi-quantify the biofilm formed on the rocks, a crystal violet assay was performed (Figure 3H, Supplementary Table S4). In the absence of the rocks, *S. desiccabilis* OD_570_ was 0.542 ± 0.017, highlighting the well-known microorganism’s ability to produce extrapolymeric substances regardless of the presence of a substrate (Reddy and Garcia-Pichel, 2007). All the rock samples, except for CC1 (probably because of larger variability), reported significantly lower mean OD_570_ values (*t*-test *p*-values < 0.05) compared to the no-rock sample. OD_570_ values were 0.141 ± 0.008 for 05AV08, 0.163 ± 0.045 for 05AV30, 0.137 ± 0.025 for BAS, 0.118 ± 0.007 for CA4, 0.220 ± 0.105 for CC1, 0.124 ± 0.016 for EU, and 0.101 ± 0.036 for L11. Non-biology controls consistently showed negligible OD_570_ values (≤0.011), confirming the absence of abiotic interference with the measurements.

### 3.4 Bioleaching from different rock substrates with *S. desiccabilis*

**Figure 4:**
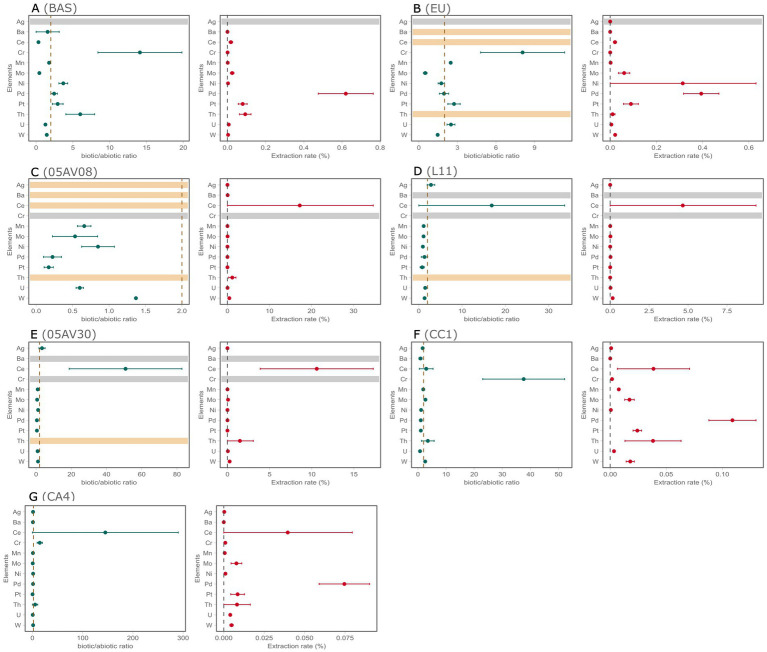
Elemental bioleaching from the 7 rock substrates by *S. desiccabilis*. A subset of 12 elements, compared to the total measured in this work, are represented here. For each rock substrate **(A–G)**, bioleaching rates are expressed as ratio values between the biotic and the abiotic extraction (biotic/abiotic ratio, left panels) and as percentage biological extraction from the rock (extraction rate, right panels). Circles represent mean values; error bars represent standard errors. In biotic/abiotic ratio panels, the vertical dotted line indicates ratio = 2, arbitrarily chosen to discriminate a positive effect of biotic over abiotic leaching. In the extraction rate panels, the vertical dotted line represents value = 0, indicating no bioleaching. Gray horizontal lines represent absence of that element in the rock substrate; yellow horizontal lines represent lack of ratio value due to absence of either biotic or abiotic leaching. Data for Fe are plotted separately (Supplementary Figure S1) due to their substantially different scale.

The effect of *S. desiccabilis* on elemental extraction from the rock substrates has been measured by ICP-MS and ICP-OES of both the rock substrates and the liquid fraction of the cultures. From this dataset, the biological efficiency of elemental extraction from the rock was evaluated by (1) elemental extraction rates (Figure 4, Supplementary Figure S1, Supplementary Table S5, Supplementary Data), and (2) biotic/abiotic extraction rates (biotic/abiotic ratio; Figure 4, Supplementary Figure S1, Supplementary Table S6, Supplementary Data). The elements that were absent in the original substrate (Supplementary Table S1, Supplementary Data) were excluded from the analysis. A summary table of the bioleaching results is reported in Supplementary Table S7.

In the BAS samples (Figure 4A, Supplementary Figure S1, Supplementary Tables S5–S7, Supplementary Data), *S. desiccabilis* promoted a small mobilization of Th [(9.31 ± 3.03) × 10^−2^%], Mo [(2.48 ± 0.94) × 10^−2^%] and Ce [(1.78 ± 0.93) × 10^−2^%], Pd [(6.20 ± 1.45) × 10^−1^%], Pt [(7.98 ± 2.31) × 10^−2^%]. Compared to the abiotic controls, strong biological enrichment from BAS was observed for Fe (biotic/abiotic ratio = 83.55 ± 13.01), Cr (14.11 ± 5.71), Th (6.00 ± 1.95), Ni (3.69 ± 0.60), Pt (2.93 ± 0.73), and Pd (2.45 ± 0.44).

In the EU samples (Figure 4B, Supplementary Figure S1, Supplementary Tables S5–S7, Supplementary Data), the highest bioleaching yields were detected for Ce [(2.08 ± 0.70) × 10^−2^%], Mo [(6.04 ± 2.44) × 10^−2^%], Ni [(3.14 ± 3.17) × 10^−1^%], Pd [(3.94 ± 0.77) × 10^−1^%], Pt [(8.98 ± 3.20) × 10^−2^%], Sr. [(1.09 ± 0.01) × 10^−2^%], W [(2.15 ± 0.32) × 10^−2^%], and Th [(1.05 ± 1.05) × 10^−2^%]. Compared to the abiotic controls, microbial activity promoted Cr (biotic/abiotic ratio = 8.07 ± 3.27), Fe (10.28 ± 2.67), Mn (2.48 ± 0.07), Pt (2.47 ± 0.48), and U (2.50 ± 0.31).

For 05AV08 (Figure 4C, Supplementary Figure S1, Supplementary Tables S5–S7, Supplementary Data), bioleaching released Ce (17.21 ± 17.51%), Th (1.15 ± 0.87%), Ba[(4.26 ± 6.03) × 10^−2^%], U [(1.51 ± 0.45) × 10^−2^%], W [(4.44 ± 1.35) × 10^−1^%], Ti [(3.11 ± 0.27) × 10^−1^%], Sr. [(1.78 ± 0.38) × 10^−1^%], and Mo [(2.80 × 1.68) × 10^−2^%]. The effect of the microbe on bioleaching was strong for Fe (50.22 ± 36.30).

In L11 (Figure 4D, Supplementary Figure S1, Supplementary Tables S5–S7, Supplementary Data), the most abundant mobilization occurred for Ce (4.66 ± 4.70%), Al [(3.41 ± 0.49) × 10^−2^%], Mo [(1.53 ± 0.33) × 10^−2^%], B [51.80 ± 20.01%], Pd [(2.22 ± 1.24) × 10^−2^%], Sr. [(1.94 ± 0.31) × 10^−1^%], Ti [(2.50 ± 0.10) × 10^−1^%], and U [(2.19 ± 0.75) × 10^−2^%]. In L11, microbial activity enhanced the extraction of Ag (biotic/abiotic ratio = 2.81 ± 0.88), Ce (16.85 ± 16.36), Fe (11.17 ± 5.20).

For 05AV30 (Figure 4E, Supplementary Figure S1, Supplementary Tables S5–S7, Supplementary Data), microbial activity led to mobilization of Ce (10.58 ± 6.71%), Th (1.48 ± 1.59%), Al [(1.33 ± 0.21) × 10^−2^ ± %], Sr. [(1.14 ± 0.36) × 10^−1^%], Mo [(6.96 ± 2.44) × 10^−2^%], U [(5.02 ± 2.32) × 10^−2^%], W [(2.57 ± 0.14) × 10^−1^%], and Ti [(3.54 ± 0.31) × 10^−1^%]. Compared to abiotic controls, strong microbial effects were evident for Ce (biotic/abiotic ratio = 50.81 ± 32.03), Fe (37.91 ± 15.80), and Ag (3.35 ± 1.75).

The most extracted elements in the CC1 (Figure 4F, Supplementary Figure S1, Supplementary Tables S5–S7, Supplementary Data) sample were Ce [(3.87 ± 3.22) × 10^−2^%], Mo [(1.72 ± 0.43) × 10^−2^%], Pd [(1.09 ± 0.21) × 10^−1^%], Pt [(2.42 ± 0.38) × 10^−2^%], Sr. [(1.15 ± 0.35) × 10^−2^%], W [(1.79 ± 0.37) × 10^−2^%], and Th [(3.82 ± 2.50) × 10^−2^%]. Strong bioleaching ratios were recorded for Cr (37.61 ± 14.57), Fe (43.12 ± 16.94), Th (3.52 ± 2.25), W (2.58 ± 0.42), Mo (2.66 ± 0.49), and Ce (2.94 ± 2.43).

Ce [(3.96 ± 3.99) × 10^−2^%], Pd [(7.46 ± 1.56) × 10^−2^%], Ru [(2.20 ± 1.19) × 10^−2^%], were the most abundant extracted element in the CA4 sample (Figure 4G, Supplementary Figure S1, Supplementary Tables S5–S7, Supplementary Data). Very strong microbial effects were observed for Ce (145.15 ± 145.15), Fe (57.60 ± 23.86), Cr (14.69 ± 5.27), and Th (5.33 ± 5.33). We note the high variability for Ce and Th, due to the fact that only one of the three experimental replicates showed concentration of these elements.

As pH is often reported as an important parameter in bioleaching efficiency, the pH of the cultures was measured after 30 days of incubation for all samples (Supplementary Table S8). While *S. desiccabilis* slightly lowered the pH of the medium even in the absence of the rock after 30 days (6.86 ± 0.03 compared to 7.01 of fresh medium), the combination of the microorganisms with some of the rock further lowered the pH, although all final values were still circumneutral. BAS (6.57 ± 0.01, *t*-test *p*-value with the no-rock control 0.0003), EU (6.61 ± 0.02; *p*-value = 0.00005), 05AV08 (6.59 ± 0.02; *p*-value = 0.00003), CC1 (6.62 ± 0.05; *p*-value = 0.005) and CA4 (6.63 ± 0.03; *p*-value = 0.0004) exhibited a significative lower pH compared to *S. desiccabilis* grown in the absence of the rock. On the other hand, L11 (6.76 ± 0.12) and 05AV30 (6.70 ± 0.10) did not change the pH compared to the no-rock control (*p*-values > 0.05). In the absence of the bacterium, rocks reported pH values between 6.94 and 7.01, similar to those of the fresh medium, indicating the rock alone did not alter the pH.

## 4 Discussion

We examined *S. desiccabilis* interactions with 7 space-relevant rock substrates, including extraterrestrial and terrestrial ones (Table 1), to assess its overall bioleaching efficiency, and thus providing a further understanding of its capabilities.

Our results indicated that *S. desiccabilis* modestly contributed to elemental bioleaching in a selective and substrate-dependent manner (Supplementary Table S7), in agreement with the current literature (Cockell et al., 2020; Santomartino et al., 2022, 2024; Tonietti et al., 2023; Plante et al., 2025). *S. desiccabilis* could bioleach metals of notable industrial relevance, namely Ce (a REE), Th, Mo, Pd, Pt (2 platinum group elements, PGEs) and W. However, microbial effects were not homogeneous across rocks, but highly dependent on the substrate. For instance, *S. desiccabilis* could extract the PGEs Pd and Pt from BAS and EU, which only contained these elements in the order of 10^−3^ ppm, but not other rocks with higher elemental concentration such as AV08 and AV30. On the other hand, Ce, Mo, and W were extracted from the McCreedy and Creighton mine rocks, but not others, despite the similar elemental concentration. The substrate-dependent differences in extraction patterns can be interpreted in function of the mineralogical composition of each rock (Figure 1), rather than elemental abundance. 05AV08 and 05AV30 are sulfide-rich substrates which contain chalcopyrite, pyrrhotite and pentlandite, minerals which are better dissolved by chemolithotrophic bioleaching mechanisms (Vera et al., 2022; Farías et al., 2021; Kaksonen et al., 2020), while BAS and EU are Si-dominated matrices composed primarily of plagioclase, pyroxene and olivine, minerals that can be preferentially leached by heterotrophic microorganisms (Schippers et al., 2013; Santomartino et al., 2022). In these substrates, metal release might be restricted to trace-element-bearing accessory phases rather than bulk silicate dissolution.

The most interesting case was the REE Ce, whose extraction from 05AV30, CC1, CA4, and L11 was enhanced by the presence of the bacterium, with extraction rates reaching 17.2% for 05AV08, although variability was high. Mean Ce bioextraction yield from BAS obtained here (1.78 × 10^−2^%) was similar to that reported in the ISS BioRock experiment, where the same rock was employed (1.44–2.84 × 10^−2^%; Cockell et al., 2020). In both cases, we observed no biological enhancement of Ce mobilization under real terrestrial gravity, although this effect was even less evident in this work (bio/abiotic ratio 0.3 this work vs. 1.0 in BioRock). Extraction of other REEs (Eu, Gd, Ho, Er, Tm) was ~4 orders of magnitude lower than in BioRock, and several were not solubilized. Such differences likely reflect the substantial methodological variation between the two experiments, including the use of minimal vs. rich medium, much lower pulp density (1% vs. 38% v/w), crushed rather than contiguous BAS, and a different incubation period (30 vs. 21 days). These factors limit direct comparison but also suggest that nutrient availability may strongly constrain *S. desiccabilis* bioleaching. Consistently with this hypothesis, a *Sphingomonas* sp. was able to remove 0.81% of Mn from electronic waste in rich medium (PDB), compared to 0.02% in carbon-enriched M9 (Huerta-Rosas et al., 2020). Similar nutrient-dependence is well documented for other heterotrophic bioleaching mechanism, for instance to produce biolixiviant (Thompson et al., 2018), providing potential insights on the underlying mechanisms for *S. desiccabilis*-mediated bioleaching that should be explored in future work. Th was also microbially enhanced in BAS and CC1, reaching extraction values between 0.09 and 1.48%. Th is a redox-inactive radionuclide with no established biological function (Hay Mele et al., 2023), whose extraction often represents a byproduct of REEs processing in industrial mining (Larrinaga et al., 2024), and that is emerging as a promising alternative to uranium in the energy sector (Atamanova et al., 2025). Due to its biological toxicity, our results pose the basis for further studies focused on the use of *S. desiccabilis* for bioremediation purposes. Consistently, tolerance to Ag, Sr., Al, Ba, and Ni has been reported for other *Sphingomonas* spp., included isolated from mining tailings (Huerta-Rosas et al., 2020; Argumedo-Delira et al., 2023).

The microbial contribution to bioleaching emerged most clearly for Fe (Supplementary Figure S1), for which biotic/abiotic ratios ranging from 10.3- to 83.5-fold were observed across all substrates, and Cr (when present in the substrate, Figure 4), with biotic/abiotic ratios ranging from 8.1 to 37.6, although their percentage of extraction was negligible in all cases. This high-ratio/low-yield combination suggests microbe-controlled trace fluxes from accessory Fe-Cr bearing minerals. For all the other elements, results were variable, with many of them reporting either effective bioleaching or relevant percentage of the extraction, but not both. For instance, U, Mo, Ni, Mn, and Ag were better leached in the presence of the bacterium from several substrates, but their % extraction is quite low. This suggests that, while *S. desiccabilis* had the potential to support the leaching of these useful metals, its economic viability varies with the substrate and needs to be carefully assessed and improved. Notably, bioleaching patterns were quite similar for the terrestrial substrate BAS and the extraterrestrial rock EU, which shared a very similar mineralogy. Measurable elemental release was also observed in abiotic controls, indicating that a fraction of dissolution and desorption occurred without biological activity. The abovementioned comparison with the BioRock results, where similar circum-neutral conditions were present but the substrate was left intact, suggests that rock crushing could have enhanced the abiotic dissolution by increasing surface area. Because this would be expected to benefit both biotic and abiotic extraction, these observations are consistent with the hypothesis that nutrient availability exerts strong control on microbial leaching. This has implications for space biomining, where the high energy cost of mechanical milling (Petersen, 2023) to achieve efficient abiotic leaching may be prohibitive. Under such constraints, employing microbial species capable of mobilizing elements without extensive grinding could offer a significant advantage (Santomartino et al., 2022).

Silicate- and oxide-dominated matrices, i.e., plagioclase, pyroxenes, olivine; feldspar and quartz, are often resistant to dissolution at circum-neutral pH, as proton-promoted weathering reactions are strongly pH-dependent (Burns, 1993). As a result, bulk mineral dissolution is not expected under the experimental conditions employed here. This kinetic limitation provides a further explanation to the low overall extraction percentages, and supports the interpretation that mobilization primarily affected trace or accessory mineral phases rather than dominant silicate matrices. This supports the hypothesis of a substrate-dependent selectivity for *S. desiccabilis*, which would not induce bulk mineral dissolution, but rather promote localized element mobilization at the microbe-mineral interface from minerals hosting rare phases (Ce and Th). Compared to acidophilic biomining systems (Rawlings and Johnson, 2007; Arshadi and Yaghmaei, 2020), bulk Fe cycling is not expected to dominate under circum-neutral and heterotrophic conditions. Previous studies have shown that biofilm-associated micro-environments (e.g., EPS-mediated complexation, enzymatically facilitated redox transformations, and micro-scale chemical gradients) can sustain localized redox gradients and promote metal mobilization even when bulk solution chemistry remains relatively stable (Bindschedler et al., 2017; Hay Mele et al., 2023), indicating a potential explanation for the consistent enrichment of Fe and Cr in the presence of *S. desiccabilis* despite the circum-neutral pH. Similar low-yield but selective mobilization patterns have been described for heterotrophic bioleaching systems targeting trace elements in silicate-rich substrates (Chaerun et al., 2017; Gumulya et al., 2022), as well as for Ag and Mn extraction from electronic waste mediated by *Sphingomonas* spp. (Díaz-Martínez et al., 2019). *S. desiccabilis* formed biofilms on all rocks, although patterns changed with the substrate, suggesting the exposed minerals could have influenced biofilm formation and bioleaching efficiency. Dense or spatially structured biofilms may enhance localized mobilization of trace elements via complexation and micro-redox chemistry, while simultaneously reflecting stress responses to toxic metal exposure. Similar links between metal stress, EPS production, and biofilm-mediated mineral interaction have been described in biomining, where EPS can buffer toxicity and concentrate metals within the biofilm matrix, promoting selective mobilization without bulk dissolution (Orell et al., 2010; Bindschedler et al., 2017; Rawlings and Johnson, 2007).

For most rocks (BAS, EU, AV08, AV30), discrepancies were observed between OD_600_ measurements and CFU-based viability. These are expected, as they account for different biological information. Optical density reflects bulk turbidity and may be influenced by cell aggregation (the effect of suspended rock particles was excluded using abiotic controls), while CFU quantification measures culturable viable cells collected from the planktonic portion of the culture. 05AV30 reduced cell viability without affecting optical density. One possible explanation is that any negative effect on microbial survival or culturability (Gadd, 2010; Maertens et al., 2021) manifested at a later stage of the growth curve, allowing normal biomass accumulation but resulting in a subsequent decline in CFU formation. This hypothesis should be tested in future time-resolved experiments monitoring microbial response throughout growth. However, results were consistent for 3 rocks. For CC1 and CA4 (both Creighton Mine rocks), both OD_600_ and CFU/mL measurements were consistently lower than in no-rock cultures, indicating that these substrates negatively affected microbial growth and viability, potentially due to the release of toxic elements (e.g., Th). *Vice versa*, L11 did not reduce either OD_600_ or CFU/mL, suggesting that this rock was not detrimental to microbial growth. Importantly, no rock completely inhibited microbial growth, with cell concentration reaching a minimum of (5.20 ± 0.34) × 10^7^ in CC1. Notably, bioleaching of various elements, including Ce and Th, still occurred on substrates where viability was reduced, indicating this did not impact bioleaching.

The extraction efficiencies remained suboptimal across all substrates, which can be explained by multiple limiting factors: (1) the static incubation, (2) the circum-neutral pH, which might constrains the kinetics of silicate and oxide dissolution, (3) diffusion-limitations at the microbe-mineral interface; (4) poor accessibility of trace elements, (5) limited nutrient availability, a condition intentionally imposed to stimulate microbial nutrient acquisition from the substrate and to replicate the resource-restricted conditions anticipated in future space settings. Although nutrient limitation may represent a critical constraint in future space biomining, particularly for heterotroph-based bioleaching, this could be mitigated through integration of biomining reactors into a closed-loop system, as previously proposed (Santomartino et al., 2022, 2023). For some *Sphingomonas* sp., metal biomass sorption has been reported as a potential bioleaching mechanism (Tangaromsuk et al., 2002; Wang et al., 2010). However, the available evidence is limited, and this mechanism does not align well with findings from other studies (Huerta-Rosas et al., 2020; Argumedo-Delira et al., 2023), including results from the BioRock experiment, in which the metals associated with *S. desiccabilis* biomass consistently accounted for <5% of the total extracted elements across gravity conditions (Cockell et al., 2020, 2021). Consequently, the dominant mechanisms driving *S. desiccabilis* bioleaching remain uncertain. Our results suggest that substrate-dependent microbe-mineral interaction likely play a role, but did not allow further mechanistic resolution. Future efforts should aim to fill this knowledge gap by identifying specific mechanisms involved and testing optimal experimental strategies to increase extraction efficiency, potentially including genetic or adaptive engineering approaches. While these extraction values are significantly lower compared to industrial standards, these results are promising when considering that they were obtained under laboratory conditions with low volumes and static growth, which do not represent a classical biomining setting. Moreover, they were obtained at circum-neutral pH values, which would represent an advantage in terms of environmentally friendly bioleaching solutions. Under improved experimental conditions, higher yields could be expected.

This study aimed to investigate the potential use of *S. desiccabilis* for bioleaching from space-relevant substrates. However, real extraterrestrial settings would differ substantially from those used in this work. Extreme thermal cycling and high radiation flux on both the Moon and Mars would require bioreactors to be shielded and thermally regulated, increasing energetic costs. Low pressure, oxidative regolith chemistry, limited liquid water stability, and nutrient and energy availability represent further limiting factors (Santomartino et al., 2022, 2024; 2026; Gumulya et al., 2022). These environmental constraints would not necessarily prevent biomining, but they would shift implementation toward optimized, contained, and engineered bioprocessing systems rather than open regolith exposure, which will also comply with planetary protection requirements (Doran et al., 2024). Such optimization would increase system complexity and energy demand, factors that are critical in space applications. Regardless of the species/consortium of interest, efforts to establish space biomining technologies should move in two complementary directions: (1) testing under comprehensive planetary parameters, e.g., reduced gravity analogues, radiation exposure, lower pressure, regolith simulants, nutrient limitations and (2) bioreactor-scale optimization studies evaluating energy cost versus extraction yield.

Our results support the targeted use of *S. desiccabilis* to enrich strategic trace metals, including REEs and PGEs, from mine tailings and low-grade ores, exploiting its ability to operate at neutral pH and with reduced chemical inputs. If optimized, its capacity for extraction from low-grade substrates such as basalts represents a key advantage for both terrestrial and space application. On Earth, it could help reduce the environmental impact associated with conventional extraction practices (Eggert et al., 2016). In space, it may enable ISRU for future settlements on Moon or Mars, where low-grade, basaltic and impact-altered lithologies are ubiquitous (Cockell and Santomartino, 2022) and reagents/energy are severely constrained. *S. desiccabilis* could act alone or in a consortium to liberate or enrich low-grade Fe-Cr-REE-Th reservoirs, while other organisms or abiotic processes handled bulk ion dissolution. Bioengineering also represents a promising approach to improve its extraction rate. Overall, the ability of *S. desiccabilis* to survive, colonize, and mobilize selected elements from such materials confirmed its robustness in mineral-rich but nutrient-poor settings, a property of relevance to future off-Earth as well as terrestrial operations.

## Data Availability

The original contributions presented in the study are included in the article/Supplementary material, further inquiries can be directed to the corresponding author.
