# Supplementary material for: Bioleaching of critical trace metals by Sphingomonas desiccabilis: substrate-driven selectivity in Earth and space analogues
Source: Front Microbiol. 2026 Apr 10;17:1741305. doi: 10.3389/fmicb.2026.1741305 (PMC13106364; doi:10.3389/fmicb.2026.1741305)
Supplement: Supplementary file 2 [file Data_Sheet_2.pdf]

Supplementary information

Bioleaching of critical trace metals by *Sphingomonas desiccabilis*: substrate-driven selectivity in Earth and space analogues

Luca Tonietti[1-4], Mattia Esposito[3], Mirko Leggiero[3], Fiona Bunn[1,5], Lorna J. Eades[6], Angelina Cordone[3], Louise Horsfall[5], Giovanni Covone[3], Charles S. Cockell[1], Donato Giovannelli[3,7-10], Alessandra Rotundi[2,4], Rosa Santomartino[1,11]*

[1] UKCA, School of Physics and Astronomy, University of Edinburgh, Edinburgh (UK)
[2] Universitá di Napoli Parthenope, Naples (Italy)
[3] Universitá degli Studi di Napoli "Federico II", Naples (Italy)
[4] INAF-OAC, Osservatorio Astronomico di Capodimonte, Naples (Italy)
[5] School of Biological Sciences, University of Edinburgh, Edinburgh (UK)
[6] School of Chemistry, University of Edinburgh, Edinburgh (UK)
[7] National Research Council, Institute of Marine Biological Resources and Biotechnologies, CNR-IRBIM, 60125 Ancona, (Italy)
[8] Department of Marine and Coastal Science, Rutgers University, New Brunswick, NJ 08901, (USA)
[9] Marine Chemistry & Geochemistry Department, Woods Hole Oceanographic Institution, Falmouth, MA 02543, (USA)
[10] Earth-Life Science Institute, ELSI, Tokyo Institute of Technology, Tokyo 152-8550, (Japan)
[11] Biological and Environmental Engineering, CALS, Cornell University, Ithaca (USA)
* Corresponding author: rosa.santomartino@cornell.edu

Supplementary Tables

Table S1. ICP-MS and ICP-OES elemental concentrations (ppm) of the seven different substrates. Data are shown as mean ± standard error. NA indicates absence of that element in the specific substrate.

Element	BAS	EU	AV08	L11	AV30	CC1	CA4
**Ag**	NA	NA	$(2.51 \pm 0.23)\times10^{1}$	$(1.31 \pm 0.07)\times10^{1}$	$(4.74 \pm 0.59)\times10^{1}$	$(1.86 \pm 0.20)\times10^{0}$	$(9.05 \pm 0.55)\times10^{0}$
**Al**	$(5.32 \pm 0.83)\times10^{4}$	$(4.95 \pm 0.33)\times10^{4}$	$(7.20 \pm 1.93)\times10^{1}$	$(5.74 \pm 0.22)\times10^{1}$	$(5.66 \pm 0.66)\times10^{1}$	$(1.21 \pm 0.17)\times10^{4}$	$(5.40 \pm 0.65)\times10^{4}$
**B**	NA	NA	NA	$(7.16 \pm 2.74)\times10^{-1}$	NA	$(1.62 \pm 1.62)\times10^{-1}$	NA
**Ba**	$(4.06 \pm 1.08)\times10^{1}$	$(2.73 \pm 0.21)\times10^{1}$	$(8.50 \pm 8.50)\times10^{-2}$	NA	NA	$(1.16 \pm 0.12)\times10^{2}$	$(3.46 \pm 0.34)\times10^{2}$
**Cd**	$(7.64 \pm 2.78)\times10^{0}$	$(1.36 \pm 0.10)\times10^{1}$	$(8.73 \pm 0.77)\times10^{1}$	$(6.68 \pm 0.29)\times10^{1}$	$(9.14 \pm 0.47)\times10^{1}$	$(4.87 \pm 0.57)\times10^{1}$	$(1.83 \pm 0.19)\times10^{1}$
**Ce**	$(1.64 \pm 0.45)\times10^{1}$	$(5.12 \pm 0.53)\times10^{0}$	$(2.06 \pm 0.52)\times10^{-1}$	$(2.32 \pm 0.63)\times10^{-1}$	$(4.54 \pm 0.30)\times10^{-1}$	$(1.27 \pm 0.13)\times10^{1}$	$(3.55 \pm 0.48)\times10^{1}$
**Co**	$(5.32 \pm 1.71)\times10^{1}$	$(1.31 \pm 0.11)\times10^{1}$	$(1.62 \pm 0.07)\times10^{2}$	$(8.23 \pm 0.26)\times10^{2}$	$(3.27 \pm 0.25)\times10^{2}$	$(9.08 \pm 0.46)\times10^{2}$	$(6.70 \pm 0.79)\times10^{2}$
**Cr**	$(3.43 \pm 1.00)\times10^{2}$	$(1.41 \pm 0.11)\times10^{3}$	NA	NA	NA	$(5.12 \pm 0.65)\times10^{2}$	$(3.07 \pm 0.35)\times10^{2}$
**Cu**	$(1.41 \pm 0.38)\times10^{2}$	$(3.40 \pm 1.00)\times10^{0}$	$(2.39 \pm 0.14)\times10^{5}$	$(6.43 \pm 0.51)\times10^{4}$	$(3.09 \pm 0.10)\times10^{5}$	$(4.66 \pm 0.29)\times10^{3}$	$(2.83 \pm 0.17)\times10^{4}$
**Dy**	$(2.78 \pm 0.36)\times10^{0}$	$(1.88 \pm 0.22)\times10^{0}$	$(6.83 \pm 1.62)\times10^{-3}$	$(1.76 \pm 0.50)\times10^{-2}$	$(1.75 \pm 0.06)\times10^{-2}$	$(9.98 \pm 0.96)\times10^{-1}$	$(2.20 \pm 0.22)\times10^{0}$
**Er**	$(1.56 \pm 0.14)\times10^{0}$	$(1.24 \pm 0.12)\times10^{0}$	$(3.99 \pm 0.94)\times10^{-3}$	$(1.58 \pm 0.76)\times10^{-2}$	$(9.12 \pm 1.30)\times10^{-3}$	$(7.24 \pm 0.76)\times10^{-1}$	$(1.33 \pm 0.14)\times10^{0}$
**Eu**	$(7.53 \pm 1.19)\times10^{-1}$	$(3.60 \pm 0.39)\times10^{-1}$	$(2.42 \pm 0.68)\times10^{-3}$	$(3.28 \pm 0.54)\times10^{-3}$	$(6.76 \pm 0.09)\times10^{-3}$	$(2.44 \pm 0.27)\times10^{-1}$	$(7.57 \pm 0.76)\times10^{-1}$
**Fe**	$(7.84 \pm 2.18)\times10^{4}$	$(1.28 \pm 0.06)\times10^{5}$	$(2.47 \pm 0.17)\times10^{5}$	$(3.85 \pm 0.13)\times10^{5}$	$(2.71 \pm 0.07)\times10^{5}$	$(3.20 \pm 0.29)\times10^{5}$	$(1.42 \pm 0.13)\times10^{5}$
**Gd**	$(2.41 \pm 0.34)\times10^{0}$	$(1.42 \pm 0.14)\times10^{0}$	$(9.27 \pm 2.53)\times10^{-3}$	$(1.13 \pm 0.18)\times10^{-2}$	$(2.49 \pm 0.28)\times10^{-2}$	$(8.40 \pm 0.96)\times10^{-1}$	$(2.35 \pm 0.25)\times10^{0}$
**Hf**	$(1.69 \pm 0.46)\times10^{0}$	$(3.51 \pm 0.46)\times10^{-1}$	$(2.70 \pm 0.57)\times10^{-3}$	$(1.86 \pm 1.24)\times10^{-2}$	$(5.87 \pm 1.21)\times10^{-3}$	(2.34 ± 0.19)×10-1	$(8.50 \pm 1.72)\times10^{-1}$
**Hg**	$(1.61 \pm 0.09)\times10^{-4}$	$(1.25 \pm 0.63)\times10^{-4}$	$(1.06 \pm 0.19)\times10^{-3}$	$(3.59 \pm 0.72)\times10^{-4}$	$(6.00 \pm 1.15)\times10^{-4}$	$(2.06 \pm 0.28)\times10^{-4}$	$(2.36 \pm 0.07)\times10^{-4}$
**Ho**	$(4.79 \pm 0.52)\times10^{-1}$	$(3.53 \pm 0.37)\times10^{-1}$	$(9.99 \pm 3.16)\times10^{-4}$	$(4.36 \pm 1.51)\times10^{-3}$	$(3.15 \pm 0.60)\times10^{-3}$	$(1.95 \pm 0.18)\times10^{-1}$	$(3.91 \pm 0.37)\times10^{-1}$
**Ir**	$(2.49 \pm 0.85)\times10^{-5}$	$(4.60 \pm 0.48)\times10^{-6}$	$(2.52 \pm 0.66)\times10^{-6}$	$(1.00 \pm 0.35)\times10^{-5}$	$(5.75 \pm 1.80)\times10^{-6}$	$(1.10 \pm 0.13)\times10^{-5}$	$(4.71 \pm 0.74)\times10^{-5}$
**La**	$(3.93 \pm 0.66)\times10^{0}$	$(1.36 \pm 0.13)\times10^{0}$	$(6.74 \pm 1.78)\times10^{-2}$	$(6.81 \pm 1.92)\times10^{-2}$	$(1.64 \pm 0.07)\times10^{-1}$	$(4.15 \pm 0.38)\times10^{0}$	$(1.37 \pm 0.20)\times10^{1}$
**Mn**	$(1.95 \pm 0.56)\times10^{3}$	$(5.92 \pm 0.32)\times10^{3}$	$(10.00 \pm 0.54)\times10^{2}$	$(3.80 \pm 0.18)\times10^{2}$	$(9.18 \pm 0.34)\times10^{1}$	$(4.50 \pm 0.43)\times10^{2}$	$(1.39 \pm 0.15)\times10^{3}$
**Mo**	$(2.06 \pm 0.57)\times10^{-1}$	$(8.82 \pm 2.55)\times10^{-2}$	$(1.28 \pm 0.20)\times10^{-1}$	$(5.76 \pm 0.49)\times10^{-1}$	$(6.28 \pm 0.30)\times10^{-2}$	$(1.44 \pm 0.24)\times10^{0}$	$(8.00 \pm 3.37)\times10^{-1}$
**Nd**	$(9.38 \pm 1.64)\times10^{0}$	$(3.97 \pm 0.36)\times10^{0}$	$(8.43 \pm 1.87)\times10^{-2}$	$(9.45 \pm 1.93)\times10^{-2}$	$(2.04 \pm 0.16)\times10^{-1}$	$(5.31 \pm 0.38)\times10^{0}$	$(1.75 \pm 0.18)\times10^{1}$
**Ni**	$(1.57 \pm 0.45)\times10^{2}$	$(1.46 \pm 1.46)\times10^{0}$	$(2.08 \pm 0.08)\times10^{4}$	$(4.15 \pm 0.11)\times10^{4}$	$(8.06 \pm 0.63)\times10^{3}$	$(3.85 \pm 0.20)\times10^{4}$	$(3.82 \pm 0.44)\times10^{4}$
**Pb**	$(5.17 \pm 1.26)\times10^{-1}$	$(6.72 \pm 2.89)\times10^{-1}$	$(3.45 \pm 0.59)\times10^{2}$	$(6.56 \pm 0.51)\times10^{1}$	$(6.02 \pm 0.22)\times10^{1}$	$(4.83 \pm 0.22)\times10^{0}$	$(1.50 \pm 0.18)\times10^{1}$

**Pd**	(1.30 ± 0.19)×10^{-3}	(1.12 ± 0.07)×10^{-3}	(1.57 ± 0.40)×10^{-1}	(1.84 ± 0.11)×10^{-2}	(1.45 ± 0.19)×10^{-1}	(2.88 ± 0.50)×10^{-3}	(4.92 ± 0.24)×10^{-3}
**Pr**	(1.61 ± 0.26)×10^{0}	(6.35 ± 0.66)×10^{-1}	(1.78 ± 0.43)×10^{-2}	(2.05 ± 0.60)×10^{-2}	(4.05 ± 0.21)×10^{-2}	(1.14 ± 0.10)×10^{0}	(3.55 ± 0.35)×10^{0}
**Pt**	(3.55 ± 0.51)×10^{-3}	(1.77 ± 0.55)×10^{-3}	(1.54 ± 0.30)×10^{0}	(2.89 ± 0.29)×10^{-1}	(4.34 ± 1.46)×10^{0}	(4.07 ± 0.64)×10^{-3}	(2.75 ± 0.98)×10^{-2}
**Rh**	(3.34 ± 1.39)×10^{-4}	(5.98 ± 1.42)×10^{-4}	(1.74 ± 0.25)×10^{-1}	(3.90 ± 0.15)×10^{-2}	(2.22 ± 0.14)×10^{-1}	(1.70 ± 0.08)×10^{-2}	(1.97 ± 0.09)×10^{-2}
**Ru**	(1.98 ± 0.61)×10^{-4}	(4.74 ± 0.79)×10^{-5}	(2.93 ± 0.46)×10^{-3}	(2.03 ± 0.06)×10^{-3}	(3.86 ± 0.10)×10^{-3}	(2.44 ± 0.17)×10^{-2}	(3.89 ± 0.41)×10^{-3}
**Sm**	(2.04 ± 0.35)×10^{0}	(9.91 ± 0.92)×10^{-1}	(1.09 ± 0.26)×10^{-2}	(1.51 ± 0.17)×10^{-2}	(2.81 ± 0.08)×10^{-2}	(8.18 ± 0.72)×10^{-1}	(2.60 ± 0.27)×10^{0}
**Sr**	(1.53 ± 0.42)×10^{2}	(6.39 ± 0.50)×10^{1}	(8.61 ± 1.66)×10^{-1}	(1.41 ± 0.16)×10^{0}	(1.47 ± 0.39)×10^{0}	(4.58 ± 0.51)×10^{1}	(2.00 ± 0.27)×10^{2}
**Tb**	(3.77 ± 0.50)×10^{-1}	(2.41 ± 0.23)×10^{-1}	(8.68 ± 2.77)×10^{-4}	(2.16 ± 0.33)×10^{-3}	(3.42 ± 0.53)×10^{-3}	(1.29 ± 0.13)×10^{-1}	(3.18 ± 0.35)×10^{-1}
**Th**	(2.36 ± 0.04)×10^{-1}	(1.94 ± 0.23)×10^{-1}	(1.06 ± 0.24)×10^{-2}	(1.61 ± 0.95)×10^{-2}	(7.96 ± 5.72)×10^{-3}	(1.01 ± 0.13)×10^{0}	(3.18 ± 0.28)×10^{0}
**Ti**	(7.90 ± 2.43)×10^{3}	(3.21 ± 0.20)×10^{3}	(1.07 ± 0.09)×10^{1}	(1.42 ± 0.05)×10^{1}	(8.80 ± 0.76)×10^{0}	(6.92 ± 0.90)×10^{2}	(3.38 ± 0.37)×10^{3}
**Tl**	(7.88 ± 1.22)×10^{-3}	(8.79 ± 0.45)×10^{-3}	(3.90 ± 0.31)×10^{-2}	(6.71 ± 0.40)×10^{-1}	(1.59 ± 0.07)×10^{-1}	(2.90 ± 0.21)×10^{-1}	(1.00 ± 0.08)×10^{0}
**Tm**	(1.86 ± 0.15)×10^{-1}	(1.51 ± 0.14)×10^{-1}	(4.09 ± 0.87)×10^{-4}	(2.09 ± 0.80)×10^{-3}	(1.38 ± 0.61)×10^{-3}	(9.73 ± 1.03)×10^{-2}	(1.62 ± 0.15)×10^{-1}
**U**	(1.10 ± 0.34)×10^{-1}	(5.90 ± 0.73)×10^{-2}	(6.26 ± 1.77)×10^{-3}	(1.07 ± 0.31)×10^{-2}	(2.42 ± 0.93)×10^{-3}	(2.95 ± 0.33)×10^{-1}	(1.03 ± 0.14)×10^{0}
**W**	(4.31 ± 1.36)×10^{-1}	(1.07 ± 0.14)×10^{-1}	(1.03 ± 0.31)×10^{-3}	(3.01 ± 0.47)×10^{-3}	(1.12 ± 0.04)×10^{-3}	(8.85 ± 1.07)×10^{-2}	(2.49 ± 0.21)×10^{-1}
**Yb**	(1.47 ± 0.13)×10^{0}	(1.24 ± 0.13)×10^{0}	(4.34 ± 0.85)×10^{-3}	(1.63 ± 0.94)×10^{-2}	(8.18 ± 0.81)×10^{-3}	(8.35 ± 0.87)×10^{-1}	(1.32 ± 0.12)×10^{0}
**Zn**	(1.49 ± 0.45)×10^{2}	(1.98 ± 0.14)×10^{1}	(3.18 ± 0.27)×10^{3}	(1.63 ± 0.13)×10^{3}	(2.51 ± 0.15)×10^{3}	(6.86 ± 0.52)×10^{2}	(5.71 ± 0.52)×10^{2}
**Zr**	(9.45 ± 2.58)×10^{1}	(1.89 ± 0.12)×10^{1}	(7.12 ± 0.67)×10^{0}	(1.38 ± 0.02)×10^{1}	(6.99 ± 0.22)×10^{0}	(1.89 ± 0.02)×10^{1}	(4.30 ± 1.12)×10^{1}

Table S2. Microbial growth expressed as optical density (OD_{600}) measured at the end of the experiment (day 30).

**Substrate**	***S. desiccabilis* containing cultures (mean ± st. err)**	**Abiotic control**
**No rock**	0.150 ± 0.015	-
**BAS**	0.026 ± 0.006	0.011
**EU**	0.033 ± 0.005	0.010
**05AV08**	0.035 ± 0.006	0.021
**L11**	0.105 ± 0.032	0.020
**05AV30**	0.114 ± 0.029	0.024
**CC1**	0.055 ± 0.016	0.010
**CA4**	0.081 ± 0.011	0.012

Table S3. Viability assay expressed as CFU/mL, measured at the end of the experiment (day 30).

Substrate	*S. desiccabilis* containing cultures (mean ± st. err)	Abiotic control
**No rock**	(2.09 ± 0.12) x 10^8	-
**BAS**	(1.16 ± 0.38) x 10^8	0.00
**EU**	(1.48 ± 0.50) x 10^8	0.00
**05AV08**	(8.00 ± 2.97) x 10^7	0.00
**L11**	(1.02 ± 0.51) x 10^8	0.00
**05AV30**	(7.87± 0.63) x 10^7	0.00
**CC1**	(5.20 ± 0.34) x 10^7	0.00
**CA4**	(1.03 ± 0.16) x 10^8	0.00

Table S4. Biofilm measurements expressed as OD_{570} from the crystal violet assay, measured at the end of the experiment (day 30).

**Substrate**	***S. desiccabilis*** **containing cultures (mean ± st. err)**	**Abiotic control**
**No rock**	0.542 ± 0.017	-
**BAS**	0.137 ± 0.025	0.011
**EU**	0.124 ± 0.016	0.002
**05AV08**	0.141 ± 0.008	0.002
**L11**	0.101 ± 0.036	0.010
**05AV30**	0.163 ± 0.045	0.003
**CC1**	0.220 ± 0.105	0.009
**CA4**	0.118 ± 0.007	0.009

Table S5. Percentage elemental extraction rate (expressed as mean±st. err. %) of a subset of elements, indicating the % element extracted by the bacterium from each rock. Light orange cells indicate values $\geq 10^{-2}$ %. NA indicates absence of that element in the rock or absence of bioextraction (i.e., mean elemental concentration in the biological sample was 0.00 ppm).

Element	BAS	EU	AV08	L11	AV30	CC1	CA4
Ag	NA	NA	$(1.76 \pm 0.45)\times10^{-5}$	$(8.98 \pm 2.85)\times10^{-5}$	$(2.08 \pm 1.11)\times10^{-5}$	$(8.42 \pm 1.92)\times10^{-4}$	$(3.23 \pm 0.66)\times10^{-4}$
Al	$(2.09 \pm 0.38)\times10^{-4}$	$(1.44 \pm 0.29)\times10^{-4}$	$(8.21 \pm 2.29)\times10^{-3}$	$(3.41 \pm 0.49)\times10^{-2}$	$(1.33 \pm 0.21)\times10^{-2}$	$(1.90 \pm 0.46)\times10^{-4}$	$(1.17 \pm 0.14)\times10^{-4}$
B	NA	NA	NA	$(5.18 \pm 2.00)\times10^{1}$	NA	$(2.30 \pm 2.30)\times10^{2}$	NA
Ba	$(5.42 \pm 5.61)\times10^{-5}$	NA	$(4.26 \pm 6.03)\times10^{-2}$	NA	NA	$(5.77 \pm 3.84)\times10^{-5}$	$(1.00 \pm 1.01)\times10^{-5}$
Ce	$(1.78 \pm 0.92)\times10^{-2}$	$(2.08 \pm 0.70)\times10^{-2}$	$(1.72 \pm 1.75)\times10^{1}$	$(4.66 \pm 4.70)\times10^{0}$	$(1.06 \pm 0.67)\times10^{1}$	$(3.86 \pm 3.22)\times10^{-2}$	$(3.96 \pm 3.99)\times10^{-2}$
Co	$(1.83 \pm 0.87)\times10^{-4}$	$(4.47 \pm 1.56)\times10^{-4}$	$(7.53 \pm 2.79)\times10^{-5}$	$(7.62 \pm 1.36)\times10^{-4}$	$(6.13 \pm 2.13)\times10^{-4}$	$(9.40 \pm 2.59)\times10^{-5}$	$(9.30 \pm 1.53)\times10^{-5}$
Cr	$(7.11 \pm 3.55)\times10^{-4}$	$(1.46 \pm 0.60)\times10^{-4}$	NA	NA	NA	$(1.67 \pm 0.68)\times10^{-3}$	$(9.53 \pm 3.59)\times10^{-4}$
Cu	$(2.59 \pm 0.94)\times10^{-3}$	NA	$(4.61 \pm 1.92)\times10^{-6}$	$(1.62 \pm 1.01)\times10^{-4}$	$(5.68 \pm 0.27)\times10^{-6}$	$(9.29 \pm 4.10)\times10^{-5}$	$(1.45 \pm 0.91)\times10^{-5}$
Eu	$(9.79 \pm 5.85)\times10^{-6}$	$(2.73 \pm 0.74)\times10^{-5}$	$(2.04 \pm 1.17)\times10^{-3}$	NA	$(3.65 \pm 3.65)\times10^{-4}$	$(1.11 \pm 1.11)\times10^{-4}$	$(6.51 \pm 3.32)\times10^{-6}$
Fe	$(1.18 \pm 0.38)\times10^{-4}$	$(8.47 \pm 2.24)\times10^{-5}$	$(8.92 \pm 6.48)\times10^{-5}$	$(1.28 \pm 0.60)\times10^{-5}$	$(5.64 \pm 2.35)\times10^{-5}$	$(4.14 \pm 1.67)\times10^{-5}$	$(1.36 \pm 0.58)\times10^{-4}$
Hf	$(2.42 \pm 0.95)\times10^{-5}$	$(4.27 \pm 4.31)\times10^{-6}$	$(1.62 \pm 1.14)\times10^{-2}$	$(6.01 \pm 4.35)\times10^{-3}$	$(5.92 \pm 5.67)\times10^{-3}$	$(3.42 \pm 1.32)\times10^{-4}$	$(6.75 \pm 4.56)\times10^{-5}$
Mn	$(1.39 \pm 0.40)\times10^{-3}$	$(1.93 \pm 0.12)\times10^{-3}$	$(9.08 \pm 1.35)\times10^{-4}$	$(4.66 \pm 0.62)\times10^{-3}$	$(1.89 \pm 0.29)\times10^{-3}$	$(7.68 \pm 0.86)\times10^{-3}$	$(6.12 \pm 1.00)\times10^{-4}$
Mo	$(2.48 \pm 0.94)\times10^{-2}$	$(6.04 \pm 2.44)\times10^{-2}$	$(2.80 \pm 1.68)\times10^{-2}$	$(1.53 \pm 0.33)\times10^{-2}$	$(6.96 \pm 2.44)\times10^{-2}$	$(1.72 \pm 0.43)\times10^{-2}$	$(7.82 \pm 3.32)\times10^{-3}$
Ni	$(3.02 \pm 0.99)\times10^{-3}$	$(3.14 \pm 3.17)\times10^{-1}$	$(7.95 \pm 2.12)\times10^{-4}$	$(2.59 \pm 0.12)\times10^{-3}$	$(3.03 \pm 0.52)\times10^{-3}$	$(6.06 \pm 0.38)\times10^{-4}$	$(1.02 \pm 0.18)\times10^{-3}$
Pd	$(6.20 \pm 1.45)\times10^{-1}$	$(3.94 \pm 0.77)\times10^{-1}$	$(2.24 \pm 1.35)\times10^{-3}$	$(2.22 \pm 1.24)\times10^{-2}$	$(4.09 \pm 2.65)\times10^{-3}$	$(1.09 \pm 0.21)\times10^{-1}$	$(7.46 \pm 1.56)\times10^{-2}$
Pt	$(7.98 \pm 2.31)\times10^{-2}$	$(8.98 \pm 3.20)\times10^{-2}$	$(3.19 \pm 1.32)\times10^{-4}$	$(2.27 \pm 1.44)\times10^{-3}$	$(2.02 \pm 1.19)\times10^{-4}$	(2.42 ± 0.38)×10^-2	$(8.58 \pm 4.26)\times10^{-3}$
Sr	$(2.79 \pm 0.78)\times10^{-3}$	$(1.09 \pm 0.10)\times10^{-2}$	$(1.87 \pm 0.38)\times10^{-1}$	$(1.94 \pm 0.31)\times10^{-1}$	$(1.14 \pm 0.36)\times10^{-1}$	$(1.15 \pm 0.35)\times10^{-2}$	$(1.38 \pm 0.21)\times10^{-3}$
Th	$(9.31 \pm 3.03)\times10^{-2}$	$(1.05 \pm 1.05)\times10^{-2}$	$(1.15 \pm 0.87)\times10^{0}$	NA	$(1.48 \pm 1.59)\times10^{0}$	$(3.82 \pm 2.50)\times10^{-2}$	$(8.19 \pm 8.23)\times10^{-3}$
Ti	$(4.41 \pm 1.37)\times10^{-4}$	$(1.10 \pm 0.07)\times10^{-3}$	$(3.11 \pm 0.27)\times10^{-1}$	$(2.50 \pm 0.10)\times10^{-1}$	$(3.54 \pm 0.31)\times10^{-1}$	$(5.28 \pm 0.72)\times10^{-3}$	$(1.14 \pm 0.13)\times10^{-3}$
U	$(6.53 \pm 2.08)\times10^{-3}$	$(5.10 \pm 0.89)\times10^{-3}$	$(1.51 \pm 0.45)\times10^{-2}$	$(2.19 \pm 0.75)\times10^{-2}$	$(5.02 \pm 2.32)\times10^{-2}$	$(3.41 \pm 0.56)\times10^{-3}$	$(4.02 \pm 0.72)\times10^{-3}$
W	$(3.39 \pm 1.13)\times10^{-3}$	$(2.15 \pm 0.32)\times10^{-2}$	$(4.44 \pm 1.35)\times10^{-1}$	$(1.59 \pm 0.34)\times10^{-1}$	$(2.57 \pm 0.14)\times10^{-1}$	$(1.79 \pm 0.37)\times10^{-2}$	$(4.81 \pm 1.06)\times10^{-3}$

Table S6 Biotic/abiotic extraction rates of a subset of elements. Values represent mean ratios ± SE for each element. NA (not available) indicates absence of that element in the rock. NL (no leaching) indicates absence of biotic or abiotic extraction (i.e., mean elemental concentration in the biological or abiotic sample was 0.00 ppm). Ratios ≥ 2 are indicated in light orange.

Element	BAS	EU	AV08	L11	AV30	CC1	CA4
Ag	NA	NA	NL	$(2.81 \pm 0.88)\times10^{0}$	$(3.35 \pm 1.75)\times10^{0}$	$(1.67 \pm 0.33)\times10^{0}$	$(1.30 \pm 0.25)\times10^{0}$
Al	$(1.48 \pm 0.13)\times10^{0}$	$(1.11 \pm 0.22)\times10^{0}$	$(4.39 \pm 0.34)\times10^{-1}$	$(9.82 \pm 1.36)\times10^{-1}$	$(5.65 \pm 0.63)\times10^{-1}$	$(5.55 \pm 1.09)\times10^{-1}$	$(1.18 \pm 0.02)\times10^{0}$
B	NA	NA	NA	$(1.20 \pm 0.07)\times10^{0}$	NA	$(1.06 \pm 0.06)\times10^{0}$	NA
Ba	$(1.57 \pm 1.57)\times10^{0}$	NL	NL	NA	NA	$(9.01 \pm 5.92)\times10^{-1}$	$(1.03 \pm 1.03)\times10^{0}$
Ce	$(3.21 \pm 1.41)\times10^{-1}$	NL	NL	$(1.68 \pm 1.64)\times10^{1}$	$(5.08 \pm 3.20)\times10^{1}$	$(2.94 \pm 2.43)\times10^{0}$	$(1.45 \pm 1.45)\times10^{2}$
Co	$(2.22 \pm 0.78)\times10^{-1}$	$(2.66 \pm 0.90)\times10^{-1}$	$(3.28 \pm 1.21)\times10^{-1}$	$(7.43 \pm 1.31)\times10^{-1}$	$(6.89 \pm 2.34)\times10^{-1}$	$(3.46 \pm 0.94)\times10^{-1}$	$(1.85 \pm 0.21)\times10^{-1}$
Cr	$(1.41 \pm 0.57)\times10^{1}$	$(8.07 \pm 3.27)\times10^{0}$	NA	NA	NA	$(3.76 \pm 1.46)\times10^{1}$	$(1.47 \pm 0.53)\times10^{1}$
Fe	$(8.35 \pm 1.30)\times10^{1}$	$(1.03 \pm 0.27)\times10^{1}$	$(5.02 \pm 3.63)\times10^{1}$	$(1.12 \pm 0.52)\times10^{1}$	$(3.79 \pm 1.58)\times10^{1}$	$(4.31 \pm 1.69)\times10^{1}$	$(5.76 \pm 2.39)\times10^{1}$
Mn	$(1.76 \pm 0.08)\times10^{0}$	$(2.48 \pm 0.07)\times10^{0}$	$(6.60 \pm 0.92)\times10^{-1}$	$(1.12 \pm 0.14)\times10^{0}$	$(9.17 \pm 1.39)\times10^{-1}$	$(1.88 \pm 0.11)\times10^{0}$	$(1.05 \pm 0.13)\times10^{0}$
Mo	$(4.42 \pm 1.15)\times10^{-1}$	$(5.00 \pm 1.42)\times10^{-1}$	$(5.34 \pm 3.10)\times10^{-1}$	$(1.10 \pm 0.22)\times10^{0}$	$(5.93 \pm 2.06)\times10^{-1}$	$(2.66 \pm 0.49)\times10^{0}$	$(6.71 \pm 0.37)\times10^{-1}$
Ni	$(3.69 \pm 0.60)\times10^{0}$	$(1.75 \pm 0.25)\times10^{0}$	$(8.49 \pm 2.24)\times10^{-1}$	$(9.35 \pm 0.34)\times10^{-1}$	$(1.15 \pm 0.18)\times10^{0}$	$(1.12 \pm 0.04)\times10^{0}$	$(1.42 \pm 0.19)\times10^{0}$
Pd	$(2.45 \pm 0.44)\times10^{0}$	$(1.96 \pm 0.36)\times10^{0}$	$(2.25 \pm 1.23)\times10^{-1}$	$(1.31 \pm 0.73)\times10^{0}$	$(4.63 \pm 2.94)\times10^{-1}$	$(1.01 \pm 0.09)\times10^{0}$	$(1.26 \pm 0.26)\times10^{0}$
Pt	$(2.93 \pm 0.73)\times10^{0}$	$(2.74 \pm 0.48)\times10^{0}$	$(1.73 \pm 0.63)\times10^{-1}$	$(7.85 \pm 4.90)\times10^{-1}$	$(4.67 \pm 2.26)\times10^{-1}$	$(1.02 \pm 0.02)\times10^{0}$	$(2.72 \pm 0.94)\times10^{-1}$
Sr	$(1.11 \pm 0.04)\times10^{0}$	$(8.98 \pm 0.39)\times10^{-1}$	$(5.74 \pm 0.34)\times10^{-1}$	$(8.42 \pm 0.95)\times10^{-1}$	$(8.72 \pm 1.44)\times10^{-1}$	$(1.58 \pm 0.45)\times10^{0}$	$(7.86 \pm 0.50)\times10^{-1}$
Th	$(6.00 \pm 1.95)\times10^{0}$	NL	NL	NL	NL	$(3.52 \pm 2.25)\times10^{0}$	$(5.33 \pm 5.33)\times10^{0}$
Ti	$(1.06 \pm 0.05)\times10^{0}$	$(9.90 \pm 0.07)\times10^{-1}$	$(1.02 \pm 0.01)\times10^{0}$	$(1.05 \pm 0.03)\times10^{0}$	$(1.03 \pm 0.01)\times10^{0}$	$(1.01 \pm 0.04)\times10^{0}$	$(1.08 \pm 0.05)\times10^{0}$
U	$(1.27 \pm 0.11)\times10^{0}$	$(2.50 \pm 0.31)\times10^{0}$	$(5.99 \pm 0.51)\times10^{-1}$	$(1.47 \pm 0.26)\times10^{0}$	$(8.57 \pm 2.18)\times10^{-1}$	$(7.36 \pm 0.87)\times10^{-1}$	$(6.98 \pm 0.81)\times10^{-1}$
W	$(1.45 \pm 0.16)\times10^{0}$	$(1.47 \pm 0.10)\times10^{0}$	$(1.37 \pm 0.01)\times10^{0}$	$(1.33 \pm 0.19)\times10^{0}$	$(1.09 \pm 0.04)\times10^{0}$	$(2.58 \pm 0.42)\times10^{0}$	$(1.44 \pm 0.30)\times10^{0}$

Table S7. Summary bioleaching table of the main extracted elements. For each element. effective extraction from each substrate ("Extracted" column) and effective bioleaching ("Bioleached" column) is indicated by an X. Green cells indicate elements where both values are ticked. Grey cells indicate absence of the element in the specific substrate.

	BAS		EU		05AV08		L11		05AV30		CC1		CA4	
	Extracted	Bioleached	Extracted	Bioleached	Extracted	Bioleached	Extracted	Bioleached	Extracted	Bioleached	Extracted	Bioleached	Extracted	Bioleached
**Ag**										X				
**Ba**					X									
**Ce**	X		X		X				X	X	X	X	X	X
**Cr**		X		X								X		X
**Fe**		X		X		X				X		X		X
**Mn**				X										
**Mo**	X		X		X				X	X	X	X		
**Ni**		X	X											
**Pd**	X	X	X								X		X	
**Pt**	X	X	X	X							X			
**Sr**			X		X				X		X			
**Th**	X	X	X		X				X		X	X		X
**Ti**					X				X					
**U**				X	X				X					
**W**			X		X				X		X	X		

Table S8. pH of the liquid fraction of each for the different samples after 30 days of incubation. The pH of the fresh medium is 7.01.

	No rock	BAS	EU	05AV08	L11	05AV30	CC1	CA4
S. desiccabilis	6.86 ± 0.03	6.57 ± 0.01	6.61 ± 0.02	6.59 ± 0.02	6.76 ± 0.12	6.70 ± 0.10	6.62 ± 0.05	6.63 ± 0.03
**Abiotic**	-	6.99	6.97	6.99	6.94	7.01	7.00	7.00

Supplementary Figures

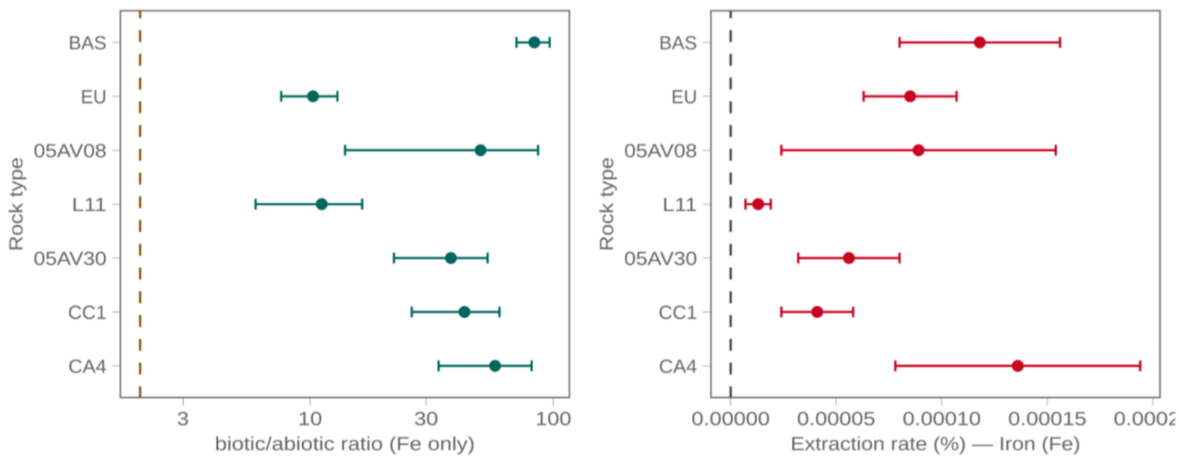

Figure S1. Bioleaching of iron (Fe) from the seven rock substrates by *S. desiccabilis*. Bioleaching rates after 30 days of incubation, expressed as ratio values between the biotic and the abiotic extraction (biotic/abiotic ratio, left panel) and as percentage biological extraction from the rock (extraction rate, right panel). In the biotic/abiotic ratio panel (left panel), the vertical dashed line represents a ratio = 2, arbitrarily chosen to indicate a positive effect of biotic over abiotic leaching. In the extraction rate panel (right panel), the vertical dashed line corresponds to a value = 0, indicating no extraction. Circles represent mean values, error bars represent standard error.

Supplementary Data

Complete bioleaching data, obtained through ICP-MS and ICP-OES.
The file contains two tabs. Tab “Sum bioleaching data” includes summary results with mean elemental concentration and standard error (ppm), calculated mean bio/abiotic ratio and standard error, and calculated extraction rate (%) and standard error. Green cells indicate biotic/abiotic ratios ≥ 2, orange cells indicate extraction rates ≥ 0.01 %.
The tab “Raw bioleaching data” reports ICP-MS and ICP-OES data for single samples. Element concentration is reported in ppm.
In both tabs, “S.desiccabilis” indicates the samples containing the microorganism, “Non-bio” indicates the abiotic cultures, and “Rock” indicates the ICP-MS/OES data for the rock substrates.
